# The impact of embodied cognition on place attachment and supportive behavior toward historic buildings in heritage sites: exploring the moderating role of resident identity climate

**DOI:** 10.3389/fpsyg.2025.1702052

**Published:** 2025-11-11

**Authors:** Shuxiang Cai, Yawen Hu, Jingyi He, Kexiang Li

**Affiliations:** 1 Institute of Fine Arts and Design, Quanzhou Normal University, Quanzhou, China; 2 Institute of Fine Arts and Design, Hunan University of Technology and Business, Changsha, China

**Keywords:** place attachment, embodied cognition, resident identity climate, historic buildings at heritage sites, cognitive-emotional-behavioral (CEB) framework, supportive behavior intention

## Abstract

The development of tourism has driven profound transformations in heritage sites, and visitors‘ place attachment is widely recognized as playing a crucial role in local sustainable conservation. However, current research remains insufficient regarding the mechanisms through which visitors' embodied cognition influences place attachment and the impact of residents‘ identity climate perceptions within this process. This study integrates embodied cognition with the Cognitive-Emotional-Behavioral (CEB) framework, introducing place attachment as a mediating variable and residents' identity climate as a moderator of the social environment. It constructs an integrated embodied cognition-emotional-behavioral-moderator (ECEB-M) model to explore how cognitions generated during tourists‘ embodied experiences influence place attachment and their supportive behavior intentions. A field survey was conducted at the historic buildings of Quanzhou, a World Heritage site, collecting 383 valid visitor questionnaires. Research findings indicate that all three dimensions of embodied cognition—multisensory perception, physical engagement, and cognitive processing—exert positive effects on place attachment and intentions toward destination-supportive behaviors. Place attachment partially mediates the relationship between embodied cognition and intentions toward destination-supportive behaviors, while also positively influencing these intentions. Resident identity climate enhanced the influence of physical engagement and cognitive processing on place attachment, positively mediating the relationship between these factors and place attachment. Resident identity climate did not produce a significant moderating effect on the relationship between multisensory perception and place attachment. Therefore, tourists' cognitive processes in forming place attachment exhibit significant differences across distinct embodied experiences. This study aims to enrich research on embodied cognition and place attachment, offering valuable insights for the protection and management of historic buildings in heritage cities.

## 1 Introduction

World Heritage sites are established under the World Heritage Convention, emphasizing the shared responsibility of governments, international organizations, and local communities to protect, present, and transmit heritage based on its Outstanding Universal Value (OUV). In recent years, the rapid growth of tourism has profoundly transformed heritage sites, with spaces undergoing rapid transformation. This has led to shifts in residents' identities and sense of place, posing challenges for heritage conservation and sustainable development (Ujang and Zakariya, 2015). Numerous World Heritage cities, such as Kyoto, Barcelona, and Dubrovnik, have experienced issues with tourist overload (Zhang and Smith, 2019), facing dual pressures from carrying capacity and governance challenges (Horlacher, 2024). Excessive commercialization and management imbalances can easily trigger risks of degradation in spatial, cultural, and community systems (Jiaxing et al., 2025; Hayes, 2020). Despite certain risks, heritage tourism remains regarded as a vital pathway for promoting cultural dissemination and economic development (Sonuç, 2023). The international community advocates for a comprehensive and responsible approach to heritage management, promoting the vitality and sustainability of heritage sites through enhanced visitor experiences, strengthened local ownership, and multi-stakeholder participation (Martínez Yáñez et al., 2022). Sustainable development goals encompass not only the preservation of physical assets but also the continuity of cultural practices and social processes (Malliet, 2017). Therefore, focusing on visitors' subjective cognition and psychological dimensions is crucial for achieving sustainable development in heritage sites.

Place attachment, as a key concept explaining the emotional bond between people and places, has been widely confirmed as a crucial psychological mechanism driving tourist behavioral intentions (Scannell and Gifford, 2010; Lewicka, 2011). It reflects tourists' unique experiences during their journeys and fosters emotional connections to destinations (Quynh et al., 2021). This emotional bond can motivate tourists to engage in positive supportive behaviors, such as eco-friendly and sustainable practices, as well as intentions to revisit and recommend the destination (Kim and Lee, 2022; Peng J. et al., 2023; Ramkissoon et al., 2012; Buonincontri et al., 2017). Consequently, place attachment is also regarded as a key factor in driving sustainable tourism development. However, existing research on place attachment has primarily focused on its robust connection to behavior, with insufficient exploration of its antecedents—particularly the specific causal mechanisms linking cognition to place attachment. Moreover, there remains a lack of systematic empirical studies examining its role within the context of heritage tourism.

The theory of embodied cognition offers a new perspective grounded in the individual level, emphasizing that cognition is not merely an abstract mental process but is closely intertwined with bodily experience (Kock and Ringberg, 2019). In heritage tourism, visitors typically enter historical architectural spaces as “presents,” interacting with the site through walking, touching, photographing, and contemplating. Through these diverse modes of engagement, they progressively construct their cognitive understanding and emotional attachment to the heritage destination (Jelić and Staničić, 2022). In recent years, research on multisensory experiences, participation, perception, memory, and meaning has garnered increasing attention. However, studies examining the influence of embodied cognition on tourist behavioral intentions remain limited. Most investigations focus on a single specific dimension (Brochado et al., 2021; Yang et al., 2022; Zhang et al., 2024b; Jelić and Staničić, 2022), with a particular lack of comprehensive analysis across different embodied dimensions. Furthermore, a systematic review of existing literature reveals that most comprehensive studies have primarily adopted qualitative exploratory approaches (Yang et al., 2022; Yin et al., 2023; Chen et al., 2025; Hu et al., 2025), with relatively limited quantitative evidence.

Moreover, as key participants in the local community, the identity of residents plays a significant role in tourists' embodied experiences. Individual tourists' emotions are influenced not only by their psychological characteristics but also by external social contexts (Hosany and Gilbert, 2010; Merleau-Ponty et al., 2013). Identity is typically understood as an individual's sense of self-recognition formed based on the characteristics of the group to which they belong (Fei et al., 1946; Sinclair-Maragh and Gursoy, 2017). It manifests as a cultural climate, constituting a crucial social environment (Chan et al., 2024). This climate may influence visitors' cognitive transformation and emotional connection (Chan et al., 2024), facilitating the generation of deep-seated experiences toward specific regions (Ujang and Zakariya, 2015). This identity climate is particularly crucial in heritage regions shaped by clan and dialect communities, as it constitutes the core element through which visitors perceive the uniqueness of the heritage (Tan and Tan, 2020). Currently, most research on resident identity adopts a resident-centered perspective, overlooking tourists' perceptions of this identity climate. Stronger resident identity often shapes more distinct cultural climate, potentially reinforcing meaning and amplifying emotions during tourists' cognitive construction processes (Lewicka, 2011). Therefore, residents' identity climate may moderate the relationship between tourists' embodied cognition and place attachment.

The Cognitive-Emotional-Behavioral (CEB) framework emphasizes the interrelationship among the cognitive, affective, and behavioral stages (Corstorphine, 2006) and has been demonstrated to explain visitor behavioral dynamics in tourism contexts effectively (Wang et al., 2020; Ren et al., 2021). Based on this, this study adopts an embodied cognition perspective, incorporating residents' identity climate as a social moderator variable into the CEB theoretical framework. It constructs an embodied cognition-emotional-behavioral-moderator (ECEB-M) conceptual model, aiming to focus on exploring the following three primary research objectives:

(1) Investigate the relationship between different dimensions of embodied cognition (multisensory perception, physical engagement, cognitive processing) and place attachment, as well as intentions toward destination-supportive behaviors.
(2) Examine the mediating role of place attachment across dimensions of embodied cognition in the relationship between place attachment and intentions toward destination-supportive behaviors.
(3) Reveal whether and how residents' identity climate mediates the relationship between the dimensions of tourists' embodied cognition and their place attachment. The findings of this study contribute to a deeper understanding of the pivotal role cognitive and psychological factors play in driving tourists' destination-supportive behavior intentions. They highlight the significant influence of different dimensions of embodied cognition and resident identity climate on the degree of place attachment, which can be effectively translated into place attachment. This provides actionable insights for the design and management of historical buildings in heritage sites, offering valuable references for the management and sustainable development of heritage tourism.

## 2 Literature review and hypothesis formulation

### 2.1 Embodied cognition-emotion-behavioral-modulation variable (ECEB-M) conceptual model

CEB originated from consumer behavior research, proposing a sequential process of “Cognition (C) - Emotion (E) - Behavior (B)” (Hirschman and Holbrook, 1986). In psychological and tourism research, extensive empirical evidence supports the chained relationship where “cognition influences emotion, and emotion directs behavior,” with emotion often serving as a key mediator (Agapito et al., 2013a; Wang et al., 2020). Research indicates that this chained relationship exhibits strong stability across different scenarios and variable configurations (Pandža Bajs, 2015; Zeithaml, 1988; Kim et al., 2013). It has been applied to explain the mechanisms underlying behavioral responses such as visitor satisfaction, sense of presence, and expectation confirmation (Corstorphine, 2006; Lin et al., 2020; Cao et al., 2022; Ren et al., 2021; Zhang and Pan, 2023; Li et al., 2025; Deng et al., 2023).

Scholars across various fields are increasingly emphasizing the adaptation and extension of theoretical frameworks in different contexts to enhance the applicability and explanatory power of models. For instance, Li et al. (2025) integrated the Expectation Confirmation Model (ECM) with the Cognitive-Emotional-Behavioral (CEB) framework in an online museum context to more comprehensively predict user behavior; Chan et al. (2024) examined the moderating role of cultural activity participation in the relationship between local cultural identity and place attachment. Although previous studies have attempted to extend or modify the CEB framework across different contexts, systematic structural adjustments tailored to heritage settings remain relatively scarce.

Based on this, this paper proposes the embodied cognition-emotional-behavioral-moderator (ECEB-M) conceptual model to enhance the explanatory and predictive validity of this framework in heritage tourism. The proposal of this model is grounded in two considerations: First, it focuses on the “cognition-emotion” link, integrating the embodied cognition perspective to conduct a more refined and systematic exploration of emotional antecedents. Furthermore, it accounts for the unique social environment of heritage sites by incorporating social moderator variables into the framework. This aims to address gaps in existing research and elucidate the role of social factors in mediating the relationship between different cognitive pathways and emotion formation. This model was proposed to enable researchers to predict changes in visitor behavior within heritage contexts more accurately and to elucidate the complex interplay between environmental cognition, psychological factors, and individual behavior.

#### 2.1.1 Theory of embodied cognition (EC)

The theory of embodied cognition proposes reexamining cognitive processes through the lens of bodily-environment interactions: cognition is not an abstract operation detached from the body (Clark, 1999; Wilson, 2002; Leitan and Chaffey, 2014; Foglia and Wilson, 2013), but instead relies on the engagement and feedback of sensory and motor systems within real-world contexts (Holland, 2004; O'regan and Noë, 2001). In human-landscape relations and spatial studies, bodily experience is defined as a prerequisite for meaning generation (Tuan, 1977; Pfeifer and Bongard, 2006; Canepa, 2022). This perspective traces back to the phenomenological tradition's discourse on “the body as the starting point of cognition” (Hilditch, 1995; Heidegger, 1977), and resonates with developmental psychology's findings on physiological maturation, contextual experiences, information processing, and sociocultural influences (Gagné, 1985; Vygotsky and Cole, 1978).

In recent years, scholars have regarded “embodiment” as a core attribute of tourism experiences, emphasizing that the interaction between the body, perception, and context is closely related to tourists' cognitive construction and emotional changes (Buonincontri et al., 2017; Xu et al., 2018; Liu and Minamikawa, 2024). For example, multisensory input and memory have been shown to enhance the experience of World Heritage sites and foster emotional connections and positive behaviors (Agapito et al., 2013b; Brochado et al., 2021; Buonincontri et al., 2017; Foglia and Wilson, 2013; Li et al., 2025). During interactions, emotional resonance, spatial ambience, and material qualities not only shape present-moment meaning construction but also extend into memory (Ujang and Zakariya, 2015; Yang et al., 2022; Jelić and Staničić, 2022), exerting lasting influence on subsequent cognition and emotion. For instance, practices such as “emotional architecture” stimulate emotional responses and meaning comprehension through spatial and narrative means (Wu and Li, 2017). Notably, interaction extends beyond physical contact to encompass social and cultural contexts (Van Dijk and Rietveld, 2017). Consequently, heritage studies have shifted focus from “material representation” to “experiential construction,” emphasizing the role of sensory engagement, interaction, and memory in value comprehension. Interdisciplinary evidence indicates that visitors generate cultural understanding through physical interaction, with heritage meanings continually interpreted and reshaped within embodied experiences (Levent and Pascual-Leone, 2014; Jelić and Staničić, 2022; Sumartojo, 2022). Architectural spaces thus transcend static “places,” exhibiting heightened performative and practical dimensions (Waterton, 2014).

Related studies typically characterize place experiences through a tripartite framework of physical, perceptual, and psychological dimensions, wherein psychological experiences driven by perception, memory, and association are regarded as key variables influencing cognitive formation (Yang et al., 2022). At the methodological framework level, existing research categorizes tourism participation into three stages: sensory, bodily, and cognitive, indicating that the first two stages form the foundation for triggering deep cognition (Yin et al., 2023). Other studies have constructed an embodied cognition paradigm for museum contexts through four dimensions—mapping, perception, context, and meaning construction (Chen et al., 2025)—and developed a cultural heritage embodied model centered on the “heritage environment–physical behavior–cognitive activity” triad (Hu et al., 2025). This evidence collectively provides sufficient grounds for this study to adopt the embodied cognition framework within the context of heritage tourism. However, existing research offers limited systematic evidence on “how historical buildings are experienced and perceived” in authentic tourism settings. Therefore, this study integrates the embodied cognition framework with the CEB framework to achieve systematic consolidation and validation, thereby revealing the operational mechanisms linking cognition, emotion, and behavior among tourists within embodied contexts.

Based on existing literature, this study refines “cognition” into three interconnected embodied dimensions tailored to the context of heritage architecture: (1) Multisensory perception: The immediate perception of heritage environments encompasses materiality, soundscapes, odors, colors, and legible cultural symbols. It also includes the overall atmosphere formed by the interplay of exhibits and derivative designs, reflecting the level of sensory engagement (Yin et al., 2023). (2) Physical engagement: The embodied interaction and presence practices of individuals with architecture and communities. This encompasses actions such as touching, photographing, and using facilities; skill experiences; participation in festivals, ceremonies, or guided tours; and other activities reflecting deep bodily involvement (Yin et al., 2023). (3) Cognitive processing: The internal integration and interpretation of sensory-motor experiences, encompassing understanding and comparison, memory and association, imagination and reflection, constitutes a critical stage in meaning construction.

#### 2.1.2 Place attachment (E) and destination-supportive behavior intention (B)

Place attachment is widely used in research examining human-place relationships (Scannell and Gifford, 2010). Scannell and Gifford (2010) proposed a three-dimensional model of place attachment encompassing cognitive, affective, and behavioral dimensions. Lewicka (2011) emphasized that place attachment is typically defined as an emotional bond between an individual and a specific location. In the context of tourism, place attachment is often referred to as destination attachment, describing the enduring emotional bond between tourists and specific destinations (Yuksel et al., 2010). Therefore, this study primarily explores the emotional dimension of place attachment. Furthermore, classic research also divides place attachment into two dimensions: place identification and place dependence (Williams et al., 1992); Subsequent research has further expanded this framework to incorporate dimensions such as social connections, lifestyle, and emotional processes, emphasizing that it encompasses both value-meaning identification and functional-activity dependence (Bricker and Kerstetter, 2000; Scannell and Gifford, 2010; Yang and Bao, 2012; Huang and Yang, 2014).

Recent reviews indicate that a relatively consistent body of evidence has emerged regarding the antecedents and consequences of place attachment (Huang and Zhang, 2024; Cai X. et al., 2025). Regarding antecedents, empirical studies reveal significant correlations between destination image, perceived value, and experience quality with place attachment (Cheng and Wu, 2015; Dlamini et al., 2021; Sahabuddin et al., 2021). At the outcome level, place attachment exhibits robust associations with two types of behavioral intentions: first, loyalty-oriented behaviors, including revisit and recommendation (Lee, 2011; Wang et al., 2020; Omo-Obas and Anning-Dorson, 2023; Kim et al., 2023); second, pro-environmental and conservation orientation, encompassing support for conservation and participation in related actions (Ramkissoon et al., 2013; Buonincontri et al., 2017; Naseri, 2020; Kim and Lee, 2022; Peng X. et al., 2023). This evidence has been replicated across diverse settings, including national parks, rural tourism destinations, historic districts, and World Heritage sites (Cheng and Wu, 2015; Cheng et al., 2021; Zhang et al., 2024a; Chan et al., 2024). For instance, in industrial heritage contexts, place attachment significantly predicts revisit intentions and ecological or conservation behavior intentions (Qiu et al., 2025; Oorgaz-Agüera et al., 2025). Therefore, this study unifies destination-related loyalty and conservation behavior intentions into a single construct termed “destination-supportive behavior intention (DSBI).”

#### 2.1.3 Identity climate (M)

Identity is typically defined as an individual's sense of self-recognition formed based on the characteristics of the group to which they belong (Fei et al., 1946; Sinclair-Maragh and Gursoy, 2017). Among these, place identity stands out, emphasizing the emotional connection between individuals and specific geographical areas, histories, and cultures (van Rekom and Go, 2006). Breakwell et al. (1986) proposed the identity process theory, suggesting that identity maintenance depends on continuity, distinctiveness, self-efficacy, and self-esteem. This theory has been applied to explain perceptions, attitudes, and behavioral intentions in tourism (Wang and Xu, 2015). Vignoles et al. (2002) further expanded this framework by incorporating “sense of belonging and meaning.”

In heritage contexts, identity is viewed as the product of long-term interactions between residents and their living environment. It is closely linked to residents' attitudes toward and support for tourism development, their sense of social responsibility, their participation in governance, and their endorsement of tourism conservation policies (Gu and Ryan, 2008; Nunkoo and Gursoy, 2012; McIlvenny et al., 2009; Gursoy et al., 2019; Qu et al., 2023; Yi et al., 2024). Heritage serves as both a symbol and source of identity (Palmer, 1999), with buildings bearing collective memories often regarded as emblems of residents' cultural identity (Rugwiji, 2019; Cittati et al., 2022). Residents construct their identity through participation in local historical traditions, cultural activities, and events (Gu and Ryan, 2008; Goulding and Domic, 2009; Zhang et al., 2019), anchoring it within tangible and intangible heritage (Mistry et al., 2015; Goulding and Domic, 2009). Residents thereby clarify their role as inheritors within the cultural heritage (Su et al., 2020). When residents position themselves as active participants rather than bystanders, their identity transforms into a stronger sense of place, thereby promoting the sustainability of local ecology and culture (Hunter, 2011). Gu and Ryan (2008) noted that pride serves as a vital source of identity; Hawke (2010) further argued that enhancing residents' sense of pride not only helps preserve local distinctiveness but also reflects the long-term stability of identity. Cittati et al. (2022) demonstrated that strengthening residents' identity contributes to better preservation of collective memory and continuity in heritage sites (Cittati et al., 2022). Additionally, residents can strengthen their identity through recognition of local significance, thereby enhancing their sense of place and community sustainability (Davis et al., 2010). Higher levels of resident identity often manifest as a more positive community climate and are accompanied by favorable evaluations (Scannell and Gifford, 2010). Therefore, in this study, we conceptualize identity as a social climate encompassing residents' perceived pride and positive evaluations, thereby reflecting the attractiveness and significance of resident identity to visitors.

In heritage tourism, visitors serve as key perceivers of the destination environment, and resident Identity is regarded as a crucial element shaping visitors' perceptions of heritage uniqueness (Tan and Tan, 2020). Resident Identity is often manifested through emotional attitudes, cultural practices, and interactions (Gu and Ryan, 2008; Goulding and Domic, 2009; Zhang et al., 2019). Collectively, these cues form a social environment that visitors can directly perceive and experience during their travel. Therefore, this study conceptualizes Identity as a social climate, uniformly termed “Identity Climate (IC).” This climate refers to the social environment formed by the outward expression of Identity and perceived by visitors, encompassing residents' sense of pride, positive evaluations, and practices, thereby reflecting its attractiveness and significance to visitors.

### 2.2 Hypothesis formulation

#### 2.2.1 The relationship between embodied cognitive and place attachment, destination-supportive behavior intention

Embodied cognition is generated through the coordinated interaction of senses, body, and mind within specific contexts (Borghi and Cimatti, 2010), while emotions are formed through the integration of perceptual and bodily responses (Niedenthal, 2007). In heritage tourism, visitors' cognitive structures form the foundation for understanding place differences and developing place attachment (Ujang and Zakariya, 2015).

First, visitors directly perceive the heritage environment through multiple sensory systems—visual, tactile, olfactory, and others—thereby forming judgments about authenticity and overall quality (Wu and Li, 2017; Liu and Minamikawa, 2024). When sensory interactions present consistent and positive cues, satisfaction and revisit intentions increase, enhancing visitors‘ perceptions of destination authenticity and subjective wellbeing (Borghi and Cimatti, 2010; Peng J. et al., 2023). Secondly, physical engagement transforms “seeing” into “being present” (Borghi and Cimatti, 2010). A satisfying visit experience helps foster place attachment among visitors (Buonincontri et al., 2017). The use of space, interactive experiences, and participatory activities enables individuals to imbue places with meaning and forge emotional connections, provided their functional needs are met and behavioral objectives supported (Williams and Roggenbuck, 1989). Furthermore, this process constitutes the primary mode of interaction between visitors and local culture. Establishing connections with the local culture helps strengthen a sense of place and cultural identity (Chan et al., 2024; Chen and Rahman, 2018). Furthermore, based on sensory and physical experiences, visitors engage in cognitive processing such as understanding, imagining, and evaluating what they see and feel (Yin et al., 2023). Cognitive processing plays a significant role in influencing tourists' behavioral intentions through meaning construction and value judgments (Buonincontri et al., 2017). Emotional, reflective, and cognitive processes during heritage site experiences not only influence immediate behavioral decisions but also exert a sustaining effect through memory recall (McIntosh, 1999). When cognitive evaluations yield positive information, they more readily trigger positive actions related to the environment (Niedenthal et al., 2001). Positive cognition also enhances the consistency between emotion and action, making behavior more executable through physical engagement (Vacharkulksemsuk and Fredrickson, 2012).

Based on the above evidence, the embodied cognition process in heritage settings can be conceptualized across three dimensions: multisensory perception, physical engagement, and cognitive processing. Consequently, this study proposes the following hypothesis in the context of heritage tourism:

H1a: Multisensory perception positively influences place attachment
H1b: Physical engagement positively influences place attachment
H1c: Cognitive processing positively influences place attachment
H2a: Multisensory perception positively influences destination-supportive behavior intention
H2b: Physical engagement positively influences destination-supportive behavior intention
H2c: Cognitive processing positively influences destination-supportive behavior intention

#### 2.2.2 The relationship between place attachment and intentions for destination-supportive behavior

In heritage contexts, place attachment is often regarded as the emotional foundation driving tourist engagement in local conservation and development. When tourists recognize the unique value of a place's spatial and cultural characteristics, they are more likely to develop attachment to it and subsequently participate in practices beneficial to the locality (Tuan, 1990). Recent research further indicates that place attachment serves as a crucial prerequisite for behavioral responses: higher attachment levels significantly increase the likelihood of developing destination-supportive behavior intentions (Shin et al., 2025). Empirical evidence also indicates that place attachment promotes more responsible local behavior and sustainable orientations (Vaske and Kobrin, 2001), while increasing the tendency to participate in local social activities (Halpenny, 2010). In other words, if tourists develop an attachment to historical buildings at heritage sites, they are more likely to engage in activities beneficial to the local community and more likely to form supportive behavior intentions toward the destination. Based on this, the study proposes the following hypothesis:

H4a: Place Attachment has a positive influence on intention toward destination-supportive behaviors.

#### 2.2.3 The mediating role of place attachment

According to the CEB theory, an individual's perception of the environment triggers emotions that influence the occurrence and transformation of environmental behaviors. The emergence of emotions occurs through the perception and evaluation of the environment, which in turn further influences an individual's behavioral intentions. Place attachment can be defined as the significant connection between an individual and a particular place—that is, the reactions generated by an individual during situational interactions. Due to the repeated association of memories and internal emotions, an attachment to a specific place develops, thereby influencing behavior (Cheng et al., 2021). Multiple empirical studies have found that place attachment plays a crucial mediating role in the relationship between tourism environmental experiences, place perception, cultural heritage revitalisation, and behavioral intentions (Cheng et al., 2021; Chan et al., 2024). Therefore, through multisensory perception, physical engagement, and cognitive processing within historic buildings, visitors form cognitive perceptions (C) of these structures, generate emotional responses (E) toward the place, and may subsequently influence supportive behavior intentions (B) related to the destination. This value transformation process aligns with the CEB theory. Based on this, the present study proposes the following hypotheses:

H3a: Place attachment mediates the relationship between multisensory perception and intention to engage in destination-supporting behaviors.
H3b: Place attachment mediates the relationship between physical participation and intention to engage in destination-supporting behaviors.
H3c: Place attachment mediates the relationship between cognitive processing and intention to engage in destination-supporting behaviors.

#### 2.2.4 The moderating role of identity climate

In heritage tourism, visitors' perceptions rely not only on direct experiences of local lifestyles, traditional customs, and built environments (Yi et al., 2017) but are also shaped by social contextual cues. As a vital component of a heritage site's distinctiveness, resident identity is perceived by visitors through presence, interaction, and contextual clues, thereby influencing their understanding and evaluation of the heritage environment. Previous studies have demonstrated that positive resident identity facilitates interactions among tourists (Chan et al., 2024) and enhances feelings of belonging and place attachment (Ujang and Zakariya, 2015). Interaction is regarded as a core element of the tourism experience, capable of transforming tourists' emotional experiences (Eusébio et al., 2018; Stylidis, 2022). Yin et al. (2023) argue that tourism interactions mediate tourists‘ cognitive evaluations and emotions during embodied participation. Empirical research indicates that the quality of interactions between tourists and residents, along with emotional intimacy, positively influences place attachment (Aleshinloye et al., 2024). Higher interaction quality correlates with deeper emotional bonds among tourists (Fan et al., 2017; Nguyen et al., 2024). The presence of residents and their interactions with tourists can influence each other's thoughts, feelings, and behaviors (Ross et al., 2010; Proshansky, 1978), prompting tourists to adjust their own behaviors through assimilation and modeling effects (Monterrubio and Mendoza-Ontiveros, 2014). McIlvenny et al. (2009) posited that the interaction between resident identity and place attachment possesses social and psychological empowerment effects, thereby influencing visitors' behavioral decisions. Further research indicates that a stronger sense of resident identity helps reinforce visitors‘ understanding of a heritage site's significance and amplifies their emotional responses (Oorgaz-Agüera et al., 2025). Furthermore, Ashforth (2000) posits that identity can serve as a motivational source for knowledge transfer, thereby strengthening individuals' attachment to a place. Driven by the desire to understand local culture, the more frequently visitors interact with residents, the stronger their yearning for more profound local experiences becomes, making them more susceptible to developing emotional attachment (Chen and Rahman, 2018).

When a place transforms, a stable identity is particularly crucial for sustaining positive perceptions of the place; its weakening inevitably undermines the place's significance (Gieryn, 2000). Understanding visitors' cognitive processes requires simultaneous consideration of embodied participation at both the individual and collective levels (Jelić and Staničić, 2022). A higher level of resident identity is often accompanied by positive evaluations of the environment and place (Scannell and Gifford, 2010). When residents express pride in their heritage, visitors may be influenced by these positive emotions. When residents actively participate in local activities, visitors may also be drawn to join in shared practices (Anton and Lawrence, 2014; Daryanto and Song, 2021). Therefore, when residents demonstrate positive attitudes toward local values and engage in corresponding practices, visitors are more likely to authentically perceive the climate shaped by identity. This may deepen their understanding and cognition of the place, potentially strengthening their sense of attachment to it.

It is worth noting that preserving heritage and historic buildings does not equate to rigidly maintaining local lifestyles. As vessels for experiential engagement, historic buildings can attract visitors to delve deeper into their cultural distinctiveness when travelers perceive a favorable identity climate through interaction. This process enhances visitors' understanding of the place, thereby strengthening their sense of place attachment. Based on this, this study proposes the following hypothesis:

H5a: The climate of resident identity perceived by visitors moderates the influence of multisensory perception on place attachment.
H5b: The climate of resident identity perceived by visitors moderates the influence of physical engagement on place attachment.
H5c: The climate of resident identity perceived by visitors moderates the influence of cognitive processing on place attachment.

### 2.3 Conceptual model

**Figure 1:**
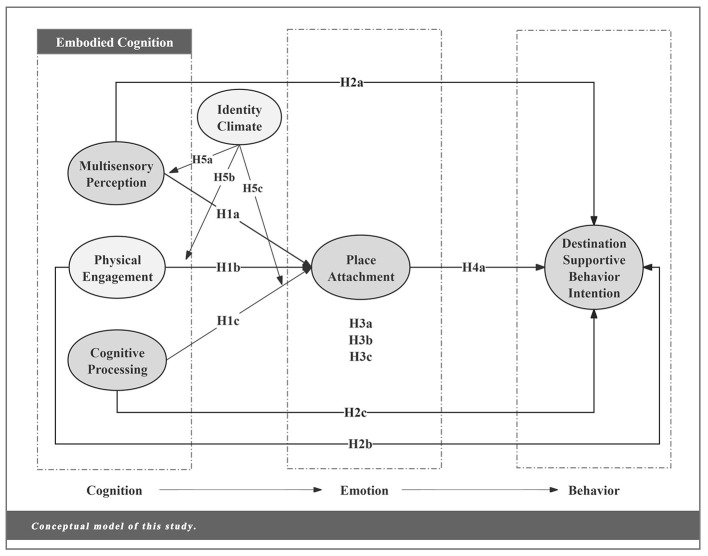
Conceptual model of this study.

This study constructs a conceptual model of ECEB-M: embodying “cognition (C)” as three dimensions—multisensory perception, physical engagement, and cognitive processing; defining “emotion (E)” as place attachment; and specifying “behavior (B)” as destination-supportive behavior intention (DSBI). Additionally, resident identity is introduced as a moderator variable, regulating the cognitive-emotional stage. The conceptual model is shown in Figure 1. Through this model, this study not only validated the effectiveness of the ECEB-M integrated model in explaining tourists' destination-supportive behavior intention but also provided more profound insights from a cognitive perspective, elucidating how the interaction between physical and social environments and psychological factors influences behavioral outcomes. Furthermore, while the model's place attachment focuses solely on the emotion dimension, its overall research logic remains consistent with Scannell and Gifford's (2010) three-dimensional model. This consistency is reflected within the CEB framework, which highlights the interconnectedness of cognitive, affective, and behavioral dimensions.

## 3 Research methods

### 3.1 Method selection

Given the nature of the research, this study adopted an appropriate methodological approach. To explore the relationships among variables within the conceptual model, structural equation modeling (SEM) was selected as the primary analytical tool. The strength of SEM lies in its ability to reveal complex relationships among latent variables simultaneously (Ringle et al., 2023). Furthermore, for variable measurement, the questionnaire survey method is widely employed due to its capacity to comprehensively reflect respondents' psychological states (Mikulić and Ryan, 2018). Consequently, this study selected the questionnaire survey method for data collection and modeled the data within the SEM framework to construct the methodological framework of this research.

This study employed partial least squares structural equation modeling (PLS-SEM) for data analysis. Although multiple platforms support PLS-SEM algorithms, SmartPLS is widely recognized for its prevalence and standardization (Dash and Paul, 2021). Therefore, PLS-SEM was selected as the data analysis platform, with SmartPLS 4.0 utilized to complete the data analysis.

### 3.2 Questionnaire design and variable selection

This study designed a preliminary questionnaire based on established scales from previous literature. The model variables in this study encompass six dimensions: multisensory perception, physical engagement, cognitive processing, identity, place attachment, and destination-supportive behavior intention. Multisensory perception was measured using scales adapted from Liu and Minamikawa (2024). Physical engagement was measured using scales based on Chen and Rahman (2018) and Jiaxing et al. (2025). Cognitive processing was measured using scales based on Chen and Rahman (2018) and Kim et al. (2012). Identity measurement draws upon scales developed by Chan et al. (2024) and Scannell and Gifford (2010). Place attachment measurement draws upon scales developed by Williams and Vaske (2003). Destination-supportive behavior intention measurement draws upon scales developed by Jiaxing et al. (2025) and Wu and Li (2017).

The questionnaire was developed in both Chinese and English, with semantic consistency ensured through back-translation (Ozolins et al., 2020). Given that this study aims to explore the relationship between tourists' cognition, emotion, and behavior within historical buildings at heritage sites, the author invited three experts from the fields of heritage tourism and architectural conservation and revitalization to review the questionnaire. They focused on evaluating the consistency of items with research variables, the clarity of wording, and the contextual relevance of content. Revisions were made based on their feedback to enhance the reliability and validity of the questionnaire.

Prior to formal distribution, researchers conducted a pilot test (*n* = 50) at the target sampling sites (Perneger et al., 2015). Based on pilot test results, questionnaire content and wording were revised to enhance readability. Following retranslation and review, the final questionnaire version was compiled—the final questionnaire comprised 27 items across six variables. Specific variable measurements and their sources are detailed in Table 1.

**Table 1:** Variable measurement items and source.

**Variable**	**Items**	**Adapted from**
Multisensory Perception (MP)	The shapes, colors, decorative patterns, and related design products in historic buildings are very attractive.	Liu and Minamikawa (2024)
	The sounds of historic buildings and their surroundings (such as voices and conversations) enhance the atmosphere of the place.	
	Smelling the scent of wood, brick, and old objects in historic buildings enhances my experience.	
	I am interested in touching traditional materials or decorative elements of historic buildings (such as brick walls, wood carvings, and stone carvings).	
	The spatial layout and atmosphere of the historic building made me feel more immersed in it.	
Physical engagement (PE)	Explanations, guided tours, and other services are very important to me.	Chen and Rahman (2018); Jiaxing et al. (2025)
	Taking photos and reading in historic buildings are important activities for me.	
	The use of facilities and technological tools (such as VR) in the space is very important to me.	
	I want to participate in local cultural activities (such as ceremonies, performances, etc.).	
	I am willing to talk with local residents or people around me.	
Cognitive processing (CP)	I am interested in learning more about the historical background and cultural significance of historic buildings.	Chen and Rahman (2018); Kim et al. (2012)
	I hope to gain a deeper understanding of the cultural elements in historic buildings, such as decorations and historical stories.	
	Understanding the customs, rituals, and lifestyles of historical buildings is important to me.	
	The more I see, hear, and feel in a space, the deeper my understanding of that culture becomes.	
	I enjoy observing the unique aspects of local cultures and find it very meaningful.	
Place attachment (PA)	The architecture of this historic city is very special to me.	Williams and Vaske (2003)
	I think this historic building is part of the local culture.	
	I really appreciate the architecture of this historic city.	
	Visiting the buildings of this historic city gave me more satisfaction than anywhere else.	
Destination-supportive behavior intention (DSBI)	If I have the opportunity, I would like to visit historical cities again.	Jiaxing et al. (2025); Wu and Li (2017)
	I would recommend this place to others.	
	I would like to participate in similar cultural activities.	
	If I have the opportunity, I am willing to support heritage preservation.	
Identity climate (IC)	The satisfaction of the local residents made me even more interested in this building.	Chan et al. (2024); Scannell and Gifford (2010)
	The pride of the local residents prompted me to learn more about this historic building.	
	By observing the local residents, I can better understand this historic building.	
	The participation of local residents gave me a deeper understanding of this building.	

The questionnaire consists of three sections. The first section uses screening questions to determine whether respondents have visited the historic building, the Official-style Mansion and stayed for at least 1 h. This criterion ensures respondents had sufficient time to familiarize themselves with the historic structures at the heritage site, fostering a degree of place attachment. This approach enhances the validity and reliability of the data collection. Part Two comprises 27 questions covering six research model variables. All items are assessed using a 7-point Likert scale, where “1” indicates strongly disagree and “7” indicates strongly agree. To minimize potential standard method bias, variables in the questionnaire are presented in random order rather than following the consistent arrangement shown in Table 1. The final section of the questionnaire consists of eight questions that gather demographic information.

### 3.3 Research subjects and sample collection

**Figure 2:**
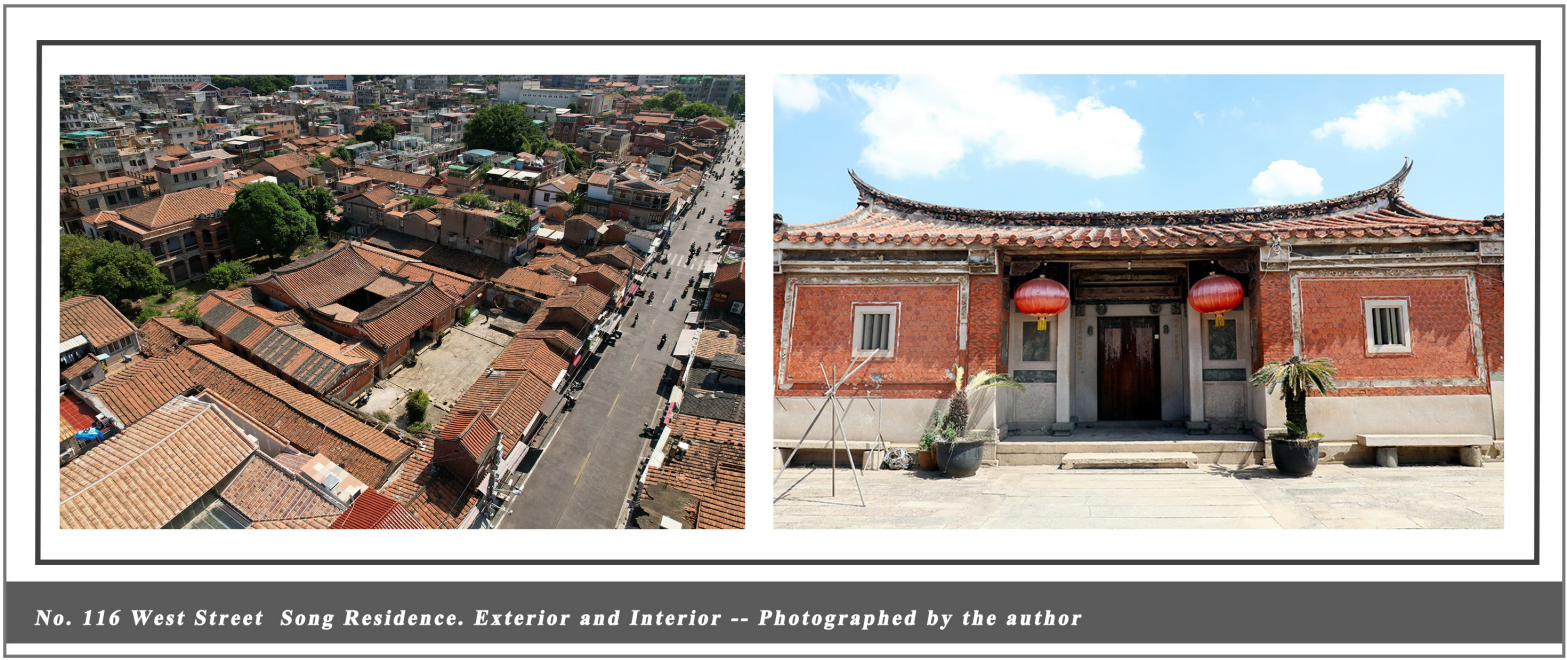
No. 481, West Street Zhuang Zheng's Former Residence. Exterior and Interior – Photographed by the author.

**Figure 3:**
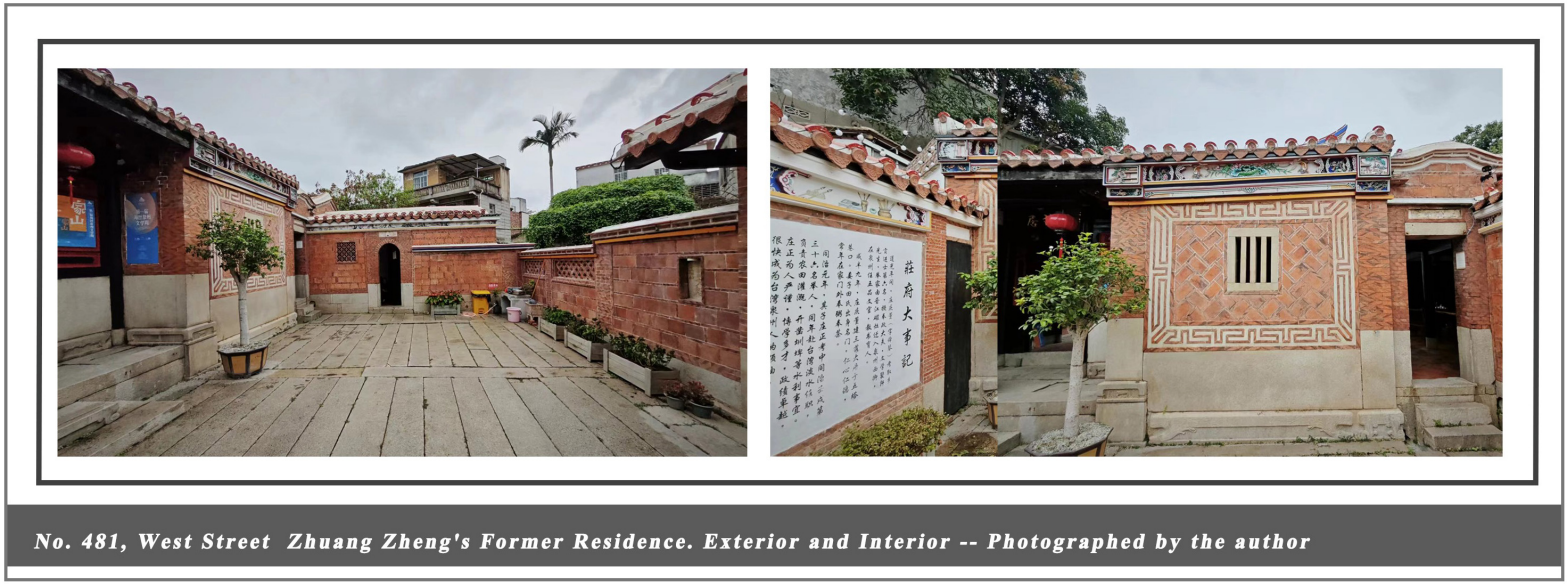
No. 116, West Street Song Residence, Exterior and Interior – Photographed by the author.

**Figure 4:**
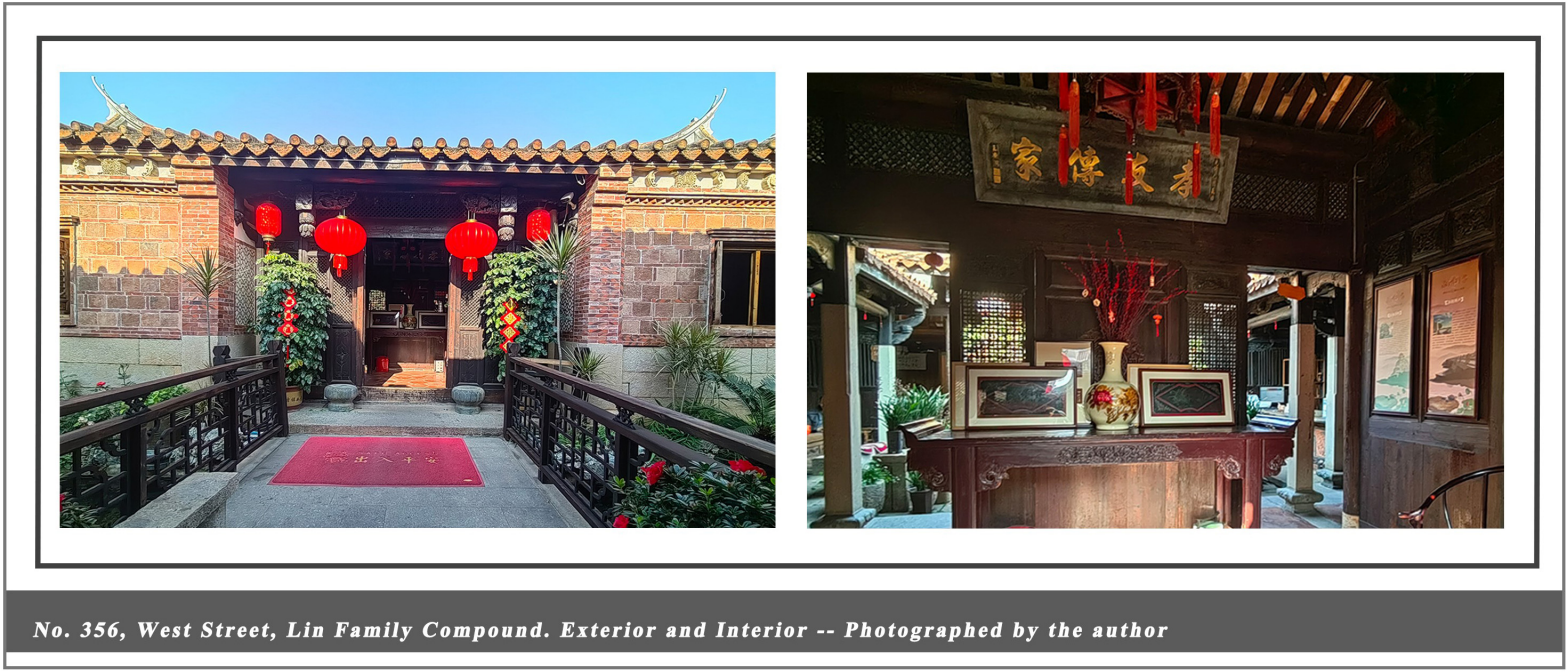
No. 356, West Street, Lin Family Compound. Exterior and Interior – Photographed by the author.

This study was conducted in the historic district of Quanzhou, where the entire 6.41-square-kilometer ancient city center is designated as a World Heritage buffer zone with well-defined spatial boundaries and comprehensive heritage documentation. The local government actively supports heritage revitalization and the integration of cultural tourism (People's Daily, 2024). In 2021, Quanzhou was inscribed on the World Heritage List as the “Maritime Metropolis of the Song and Yuan Dynasties in China.” Under this unique historical identity, Quanzhou has cultivated a historical architectural complex that blends regional characteristics with multicultural influences, carrying profound social memories and clan cultural traditions. Of particular significance is the core architectural type, the Official-style Mansion, which, as a quintessential example of traditional Chinese vernacular housing (see Figures 2–4), remains closely intertwined with the daily life of the community to this day. Driven by the “World Heritage effect” and tourism expansion, Quanzhou has witnessed a surge in visitor numbers. Annual tourist arrivals rose from 66.76 million in 2021 to surpass 100 million in 2024, with growth projected to continue into 2025 (Dongnan News, 2024). Urban environments are undergoing rapid transformation and change. Like World Heritage cities such as Kyoto and Dubrovnik, they face the dual challenges of preservation and development. This provides a rich empirical setting for the present study: high-density tourist flows, a complete and diverse array of historic buildings, and vibrant community cultures offer an exceptionally suitable research context for observing how visitors' cognitive processes during embodied experiences influence place attachment and destination-supportive behavior intentions.

This study selected the Quanzhou Official-style Mansion as its architectural case study based on the following criteria: (1) Historical and cultural representativeness (encompassing history, indigenous craftsmanship, multiculturalism, and clan culture) (2) Well-preserved and densely distributed throughout the historic district of Quanzhou (3) Primarily serving residential or revitalization purposes, these structures offer diverse experiential value encompassing architecture, landscape, and community interaction. Selecting this building type as data collection points ensures respondent representativeness, thereby enhancing the credibility of findings.

The questionnaire was distributed to visitors through researchers' personal connections and at various attractions within the historic district. To ensure the sample represented the overall visitor population, field surveys were conducted on both weekdays and weekends. This study collected a total of 405 questionnaires. Among these, 22 respondents indicated they had not yet visited historical buildings of the Official-style Mansion type. Consequently, these samples were excluded, resulting in a final count of 383 valid questionnaires. According to the “10-fold rule of thumb” for PLS-SEM (Barclay et al., 1995; Hair et al., 2019), the sample size should be at least ten times the number of measurement items for the most complex latent variable in the model or the maximum number of exogenous paths pointing to that latent variable. The adequate sample size in this study exceeds the standard, ensuring the robustness and reliability of the analytical results.

## 4 Discovery

### 4.1 Demographic information

Table 2 presents the demographic information of the survey respondents. The gender distribution was relatively balanced, with women slightly outnumbering men (51.7%). The largest age group was 25–34 years old (32.64%), followed by 35–44 years old (29.77%), indicating that the respondents were predominantly young and middle-aged adults. Respondents' places of birth were widely distributed, including local (39.43%), other provinces (32.38%), and overseas (8.62%). The largest occupational group among respondents was corporate employees (45.69%), followed by 45.69 21.67%. Regarding visit frequency, 53.26% of respondents were first-time visitors, indicating that nearly 46.74% had revisited this type of architectural site in Quanzhou, while 16.97% had visited three or more times. When learning about the site, most tourists reported discovering it through social media (44.14%) or travel platforms (17.23%). Additionally, most respondents indicated that traveling with friends (43.6%) and family (28.2%) were their primary choices. Overall, the sample structure is reasonable. Furthermore, repeat visits to the same buildings by respondents were relatively common, enhancing the validity of the place attachment assessment. Therefore, the sample possesses a certain degree of representativeness.

**Table 2:** Descriptive statistical analysis.

**Gender**	**Frequency**	**Percentage**	**Age group**	**Frequency**	**Percentage**
Male	185	48.3	18-24 years old	78	20.37
Female	198	51.7	25-34 years old	125	32.64
**Number of Visits**			35-44 years old	114	29.77
First time	204	53.26	45-54 years old	31	8.09
Second time	114	29.77	55-64 years old	22	5.74
Third time and above	65	16.97	65 years old and above	13	3.39
**Source of information**			**Mode of Travel**		
family and friends	79	20.63	Travel alone	60	15.67
Social media	169	44.13	Traveling with family	108	28.2
Travel platforms	66	17.23	Travel with friends	167	43.6
Official channels	30	7.83	Group travel	34	8.88
Spontaneous visit	39	10.18	Others	14	3.66
**Occupation**			**Education Level**		
Student	83	21.67	Elementary school and below	25	6.53
Corporate employee	175	45.69	Junior high school	38	9.92
Civil servant	32	8.36	High school/vocational school	55	14.36
Freelance	48	12.53	Undergraduate degree	182	47.52
Retirement	21	5.48	Master's degree or above	83	21.67
Others	24	6.27			
**place of birth**			**place of birth**		
Quanzhou	151	39.43	Other cities in Fujian Province	75	19.58
other provinces	124	32.38	overseas	33	8.62

### 4.2 Establishing reliability and validity

Table 3 presents the mean, standard deviation, and factor loadings for all measurement items across variables. Table 4 lists Cronbach's alpha composite reliability (CR) and average variance extracted (AVE) values. Cronbach's alpha coefficients for all variables in the model ranged from 0.824 to 0.891, while CR values ranged from 0.879 to 0.924. These standards meet the reliability criteria for estimating PLS-SEM models as defined by Gefen et al. (2000), thereby ensuring the reliability of the estimates. According to Hair et al. (2019), when Cronbach's α and CR values both exceed 0.7 and the AVE value surpasses 0.5, the data demonstrates good reliability and convergent validity.

**Table 3:** Descriptive statistics and factor loading of items.

**Items**	**Mean**	**Standard deviation**	**Skewness**	**Excess kurtosis**	**Factor loading**
MP1	5.590	1.323	−1.028	0.564	0.821
MP2	5.282	1.472	−0.941	0.360	0.791
MP3	5.405	1.371	−1.058	0.841	0.847
MP4	5.522	1.408	−0.976	0.363	0.816
MP5	5.478	1.540	−1.184	0.828	0.813
PE1	4.977	1.788	−0.708	−0.510	0.838
PE2	4.762	1.689	−0.596	−0.496	0.827
PE3	5.217	1.671	−0.871	−0.039	0.875
PE4	5.089	1.665	−0.812	−0.032	0.820
PE5	4.854	1.691	−0.615	−0.415	0.809
CP1	5.548	1.368	−1.058	0.812	0.748
CP2	5.501	1.378	−1.248	1.678	0.763
CP3	5.426	1.443	−1.219	1.298	0.762
CP4	5.431	1.394	−1.093	0.980	0.810
CP5	5.520	1.405	−1.200	1.168	0.764
PA1	5.470	1.561	−1.176	0.845	0.753
PA2	5.345	1.511	−1.088	0.728	0.814
PA3	5.264	1.530	−0.984	0.529	0.841
PA4	5.366	1.494	−1.092	0.852	0.831
DSBI1	5.225	1.573	−1.001	0.415	0.915
DSBI2	5.141	1.582	−0.883	0.124	0.867
DSBI3	5.499	1.486	−1.099	0.739	0.841
DSBI4	5.308	1.562	−1.035	0.560	0.846
IC1	5.230	1.505	−0.933	0.477	0.849
IC2	5.170	1.570	−0.980	0.516	0.810
IC3	5.120	1.537	−0.859	0.279	0.798
IC4	5.125	1.605	−0.992	0.333	0.770

**Table 4:** Reliability and convergent validity test.

**Variable**	**Cronbach's α**	**CR**	**AVE**
Multisensory perception (MP)	0.877	0.910	0.669
Physical engagement (PE)	0.891	0.920	0.696
Cognitive processing (CP)	0.828	0.879	0.593
Place attachment (PA)	0.825	0.884	0.657
Destination-supportive behavior intention (DSBI)	0.890	0.924	0.753
Identity climate (IC)	0.824	0.882	0.651

Tables 5, 6 present the results of the discriminant validity tests. According to the criteria established by Fornell and Larcker (1981) and Voorhees et al. (2016), since the square root of the average variance extracted (AVE) for each latent variable exceeds the absolute value of the correlation coefficient with any other latent variable, all variables in this study are independent of each other. This indicates that the model possesses good discriminant validity. Simultaneously, we employed the heterospecific-monospecific trait (HTMT) ratio to assess discriminant validity, setting the threshold at 0.85 as recommended by Roemer et al. (2021). Consequently, all variables in the study model demonstrated satisfactory HTMT values (0.300–0.586).

**Table 5:** Discriminant validity test.

	**Physical engagement**	**Destination-supportive behavior intention**	**Cognitive processing**	**Multisensory perception**	**Identity climate**	**Place attachment**
Physical engagement	0.834					
Destination-supportive behavior intention	0.524	0.868				
Cognitive processing	0.349	0.576	0.770			
Multisensory perception	0.248	0.400	0.446	0.818		
Identity climate	0.190	0.345	0.336	0.262	0.807	
Place attachment	0.340	0.608	0.442	0.326	0.253	0.811

**Table 6:** HTMT test.

	**Physical engagement**	**Destination-supportive behavior intention**	**Cognitive processing**	**Multisensory perception**	**Identity climate**	**Place attachment**
Physical engagement						
Destination-supportive behavior intention	0.586					
Cognitive processing	0.397	0.667				
Multisensory perception	0.273	0.444	0.533			
Identity climate	0.218	0.406	0.385	0.309		
Place attachment	0.396	0.708	0.529	0.376	0.300	

Additionally, to ensure high correlations among explanatory variables did not confound the results, multicollinearity was examined for each variable. Table 7 presents the results of the multicollinearity analysis. According to Hair et al. (2019), the variance inflation factor (VIF) values for all observed variables were less than 5, indicating no significant multicollinearity issues in the data.

**Table 7:** Collinearity assessment.

**Items**	**VIF**
MP1	2.050
MP2	1.996
MP3	2.240
MP4	2.049
MP5	1.856
PE1	2.240
PE2	2.193
PE3	2.551
PE4	2.067
PE5	2.012
CP1	1.894
CP2	1.903
CP3	1.557
CP4	2.064
CP5	1.830
PA1	1.517
PA2	1.818
PA3	1.976
PA4	1.847
DSBI1	3.285
DSBI2	2.348
DSBI3	2.187
DSBI4	2.224
IC1	1.929
IC2	1.761
IC3	2.200
IC4	2.126

### 4.3 PLS analysis results

#### 4.3.1 Direct effect analysis

Based on the research by Hair et al. (2012), this study analyzed 383 valid samples and 5,000 subsamples to assess path significance. Table 8 presents the analysis of direct effects among variables. Results indicate that cognitive processing has the strongest direct influence on place attachment (β = 0.320, *p* < 0.001). Both multisensory perception (β = 0.158, *p* < 0.05) and physical engagement (β = 0.236, *p* < 0.001) exert significant positive effects on place attachment. H1a, H1b, and H1c were supported. Regarding behavioral intention prediction, physical engagement exerted the strongest direct effect on behavioral intention (β = 0.283, *p* < 0.001). Multisensory perception (β = 0.088, *p* < 0.05), cognitive processing (β = 0.279, *p* < 0.001), and place attachment (β = 0.359, *p* < 0.001) all exhibited significant positive effects. Hypotheses H2a, H2b, H2c, and H4a were supported.

**Table 8:** PLS direct effect.

**Hypothesis**	**Path**	**Coefficients**	***T***	***P***	**Result**
H1a	Multisensory Perception → Place Attachment	0.158^**^	2.782	0.005	Support
H1b	Physical Engagement → Place Attachment	0.236^***^	4.743	0.000	Support
H1c	Cognitive Processing → Place Attachment	0.320^***^	5.568	0.000	Support
H2a	Multisensory Perception → Destination-Supportive Behavior Intention	0.088^*^	2.021	0.043	Support
H2b	Physical Engagement → Destination-Supportive Behavior Intention	0.283^***^	5.638	0.000	Support
H2c	Cognitive Processing → Destination-Supportive Behavior Intention	0.279^***^	5.693	0.000	Support
H4a	Place Attachment → Destination-Supportive Behavior Intention	0.359^***^	8.102	0.000	Support

#### 4.3.2 Indirect effects analysis

This study employed the Bootstrap method with 5,000 resampling iterations and 95% confidence intervals to test the mediating effects. If the confidence interval did not include 0, it indicated a significant effect. The results of the mediation effect test are shown in Table 9. Place attachment played a significant partial mediating role in all three paths. The indirect effect of cognitive processing through Place attachment was the largest (β = 0.115, 95% CI [0.068, 0.166]), followed by physical engagement (β = 0.085, 95% CI = [0.047, 0.132]) and multisensory perception (β = 0.057, 95% CI = [0.016, 0.101]). H3a, H3b, and H3c were supported.

**Table 9:** PLS mediating effect.

**Hypothesis**	**Path**	**Coefficients**	**Bias-Corrected**		**Result**
			**95%CI**		
H3a	Multisensory Perception → Place Attachment → Destination-Supportive Behavior Intention	0.057	0.016	0.101	Support
H3b	Physical Engagement → Place Attachment → Destination-Supportive Behavior Intention	0.085	0.047	0.132	Support
H3c	Cognitive Processing → Place Attachment → Destination-Supportive Behavior Intention	0.115	0.068	0.166	Support

#### 4.3.3 Moderation effect analysis

**Figure 5:**
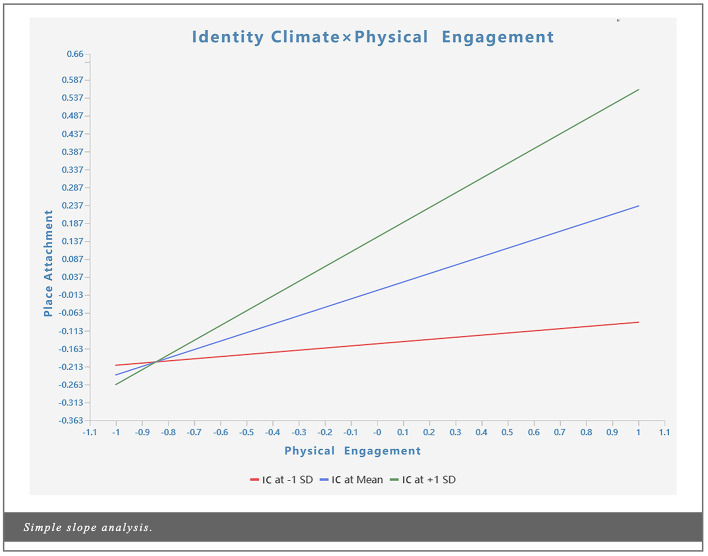
Simple slope analysis (Identity Climate × Physical Engagement).

**Figure 6:**
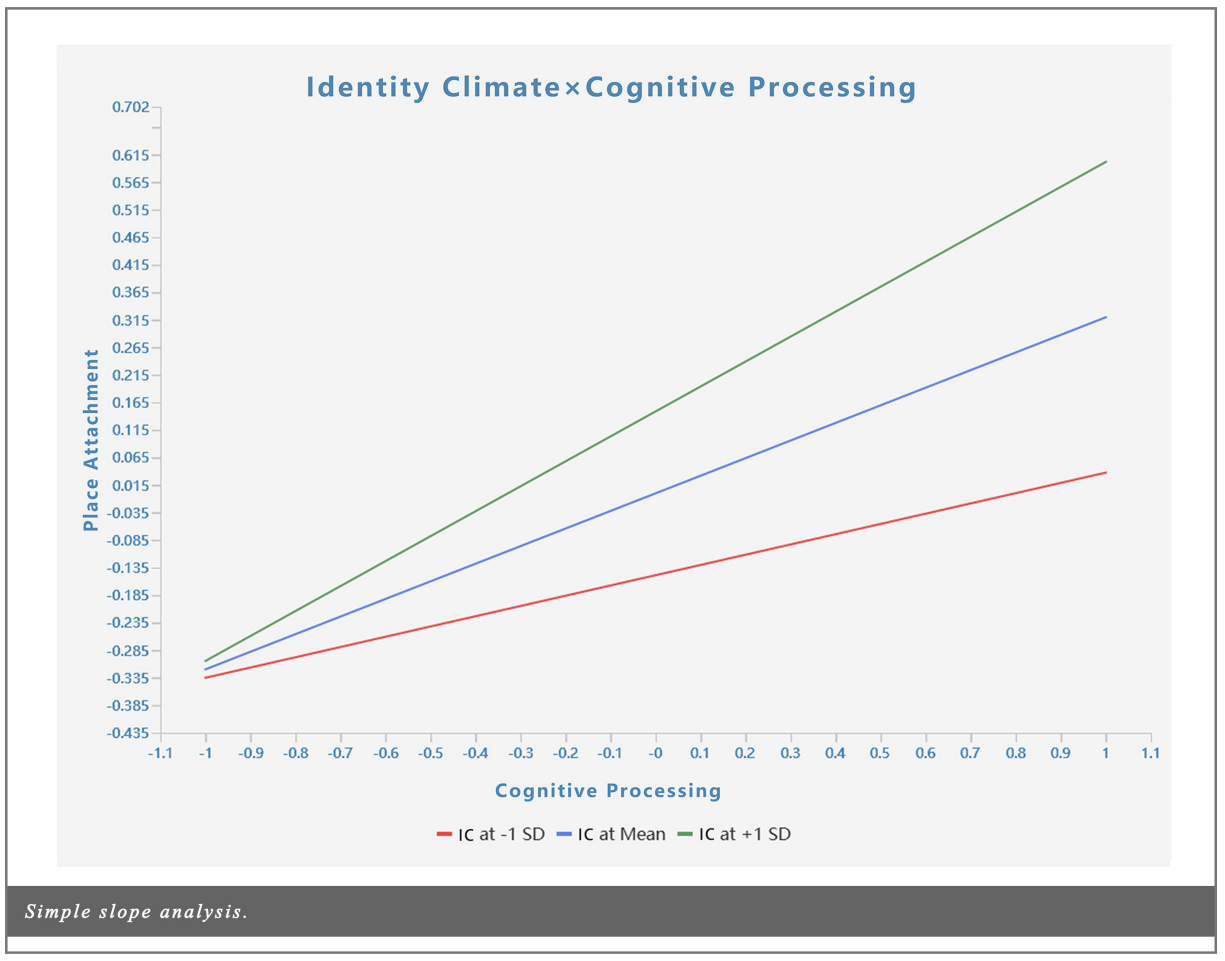
Simple slope analysis (Identity Climate × Cognitive Processing).

According to Hair et al. (2017), the significance of moderation effects is determined by whether the *p*-value of the interaction term falls below the predetermined threshold (*p* < 0.05). Table 10 presents the results of the moderation effect tests. Specifically, identity climate exerted a positive moderating effect on the relationship between place attachment and physical engagement (β = 0.176, *t* = 3.527, *p* < 0.001) as well as cognitive processing (β = 0.133, *t* = 1.980, *p* < 0.05). This implies that the higher the residents' identity climate, the more pronounced the transformation of tourists' physical engagement and cognitive processing into place attachment becomes. Therefore, H5b and H5c are supported. This finding aligns with the perspective of Ujang and Zakariya (2015), suggesting that relevant architectural managers can enhance their focus on residents. By fostering a positive identity climate, they can more effectively promote the transformation of cognition into place attachment. Figures 5, 6 present a simple slope analysis of identity climate on place attachment. When increasing the mean or subtracting one standard deviation, the effect of the interaction term between identity climate and physical engagement or cognitive processing differs significantly.

**Table 10:** PLS moderating effect.

**Hypothesis**	**Path**	**Coefficients**	***T***	***P***	**Result**
H5a	Identity Climate^*^Multisensory Perception → Place Attachment	0.026	0.385	0.700	Reject
H5b	Identity Climate^*^Physical Engagement → Place Attachment	0.176^***^	3.527	0.000	Support
H5c	Identity Climate^*^Cognitive Processing → Place Attachment	0.133^*^	1.980	0.048	Support

**Figure 7:**
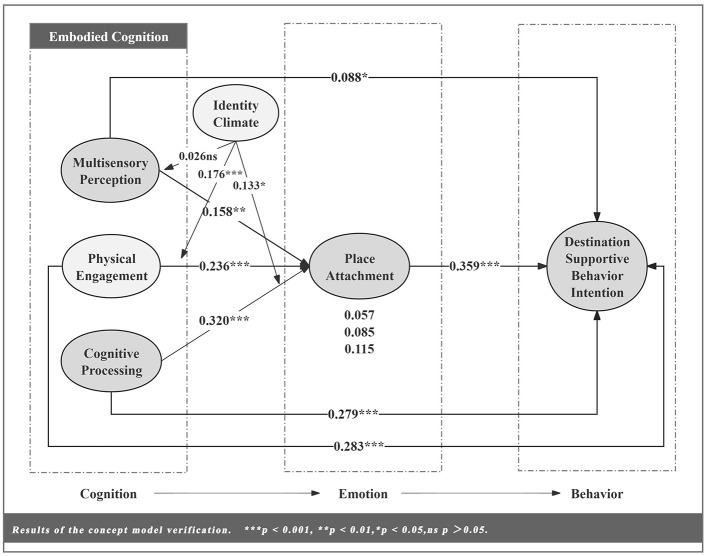
Results of the concept model verification.

However, based on prior experimental data, the moderating effect of identity climate was not confirmed in the relationship between multisensory perception and place attachment (*t* = 0.385, *p* > 0.05). This result indicates that H5a is rejected. Therefore, the influence of multisensory perception on place attachment may not be significantly affected by the moderating variable of identity climate. Figure 7 presents the final validation results of the conceptual model.

Table 11 presents the results for the model's R^2^ and Q^2^ values. According to the criteria established by (Cohen 1988) and (Rehman Khan and Yu 2021), all R^2^ values fall within the range of 0.33 to 0.67, and all Q^2^ values exceed zero. Consequently, the model demonstrates strong explanatory power and exhibits favorable predictive correlation.

**Table 11:** R square and Q square.

	***R* ^2^**	***Q* ^2^**
Destination-supportive behavior intention	0.563	0.446
Place attachment	0.360	0.308

## 5 Conclusion

This study conducted a questionnaire survey among tourists visiting historic buildings in the historic district of Quanzhou City, Fujian Province—a newly designated World Heritage site in China. Based on the ECEB-M model, “cognition” was embodied into three dimensions: multisensory perception, physical engagement, and cognitive processing. The research explored how these dimensions influence place attachment and destination-supportive behavior intention, with resident identity climate serving as a social moderator variable at the cognitive-emotion stage. The primary focus of this study is to identify the embodied antecedents of place attachment, systematically elucidate how cognition and emotion translate into positive behaviors, and examine the specific pathways through which resident identity climate, as a social factor, influences cognition and emotion.

This study validated the fundamental antecedents of place attachment among tourists in embodied experiences. First, the findings confirmed that embodied cognition exerts a positive influence on place attachment. Specifically, multisensory perception, physical engagement, and cognitive processing all showed significant positive correlations with place attachment. Effect sizes increased progressively, with cognitive processing exerting the relatively most decisive influence. In other words, compared to “seeing” and “participating,” understanding, reflecting upon, and constructing meaning around heritage more effectively transforms experiences into enduring emotional connections. This finding supports prior theoretical research (Hu et al., 2025; Yang et al., 2022; Yin et al., 2023). It also resonates with the advocacy for heritage conservation to focus on “experiential meaning generation” (Sumartojo, 2022).

Furthermore, the significant positive influence of place attachment on destination-supportive behavior intention aligns with prior research (Wang et al., 2020; Omo-Obas and Anning-Dorson, 2023; Kim et al., 2023). For instance, Peng X. et al. (2023) demonstrated place attachment's pivotal role in fostering environmentally friendly behaviors. Collectively, these studies indicate that emotion factors—such as place attachment—are crucial for promoting positive behavioral intentions among tourists.

Second, regarding the influence of embodied cognition on destination-supportive behavior intention. Results indicate that multisensory perception, physical engagement, and cognitive processing are positively correlated with behavioral intention. Among these, physical engagement exerts the most decisive influence. Through interactive activities and hands-on participation, visitors are more likely to translate cognitive insights into actionable intentions. This may stem from visitors establishing more direct connections with the destination. This supports the notion that heritage tourism emphasizes “practical participation” (Jelić and Staničić, 2022). This observation is also broadly consistent with previous research (Yin et al., 2023; Chen et al., 2025). Furthermore, while multisensory perception exerts a relatively minor yet stable influence, this indicates that material authenticity serves as a foundational condition, supporting prior research (Jiaxing et al., 2025). Conversely, the social dimension and sense of meaning within the experience play a comparatively more prominent role in driving behavior.

Third, the study found that place attachment partially mediated the relationship between multisensory perception, physical engagement, and cognitive processing on supportive behavior intention. These findings align with previous literature (Wang et al., 2020; Qiu et al., 2025; Oorgaz-Agüera et al., 2025). This indicates that the three dimensions of embodied cognition exert a more pronounced positive influence on destination-supportive behavior intention through place attachment, thereby validating the logical framework of the CEB. At the same time, this highlights the partial key role of emotional states in transforming cognition into destination-supportive behavior intention. The findings from the three pathways are supported by existing literature: The relationship between multisensory perception and destination-supportive behavior intention is mediated by place attachment, as supported by Liu and Minamikawa (2024). The relationship between physical engagement and destination-supportive behavior intention is mediated by place attachment, as supported by Jiaxing et al. (2025). Furthermore, the relationship between cognitive processing and destination-supportive behavior intention is mediated by place attachment, consistent with the findings of Buonincontri et al. (2017). This study not only re-examined these conclusions but also further expanded and confirmed the moderating role of identity climate in the place attachment pathway.

Fourth, it is noteworthy that identity climate plays a selective moderating role across the three pathways within the embodied cognition-place attachment stage. Research indicates that identity climate significantly strengthens the relationship between physical engagement, cognitive processing, and place attachment, but does not moderate the relationship between multisensory perception and place attachment. This can be interpreted as follows: when residents' pride and satisfaction with their locality are externalized into a perceptible social climate, visitors are more likely to interpret their experiences as “something worth caring about and preserving” during the engagement and comprehension stages. This, in turn, facilitates the development of attachment. For instance, resident participation may enhance visitors' interest in learning about architecture, manifesting as a greater willingness to explore the building's history and other factual information. Moreover, deepening memory and understanding, and catalyzing meaning generation, further facilitate the establishment of emotional connections between visitors and the destination. This finding aligns with previous literature (Daryanto and Song, 2021; Chen and Rahman, 2018; Aleshinloye et al., 2024). During the multisensory perception stage, however, identity climate cues exert limited influence. This may stem from the fact that at this stage, visitors‘ perceptions of the place remain confined mainly to superficial physical dimensions. This finding confirms that interactions at the sensory level are superficial and immediate (Kastenholz et al., 2013), while identity climate plays a greater role in deeper interactions and cultural engagement processes (Hunter, 2011; Kyle et al., 2005). When physical interaction and thoughtful understanding are absent, the mere coexistence of residents and visitors fails to generate emotional bonds, aligning with existing research (Nguyen et al., 2025). In other words, for residents' identity climate to truly “take effect,” connections must be forged through genuine physical interaction. This allows their identity to be seen, absorbed, and ultimately transformed into attachment through participation and understanding.

## 6 Discussion

### 6.1 Management perspective

This study examines place attachment and destination-supportive behavior intention through an analysis of tourists' embodied cognition of historical buildings at heritage sites, offering insights into the management and development of such sites.

First, multisensory perception, physical engagement, and cognitive processing contribute to enhancing visitors' attachment to a place and their supportive behavior intentions. Heritage site management and conservation personnel should focus on optimizing the experiential interactivity of spatial design while ensuring the authenticity and protective integrity of the architectural fabric. This involves providing opportunities for visitors to engage deeply through interactive guided tours, traditional ceremonies, intangible cultural heritage craft experiences, and similar activities. At the same time, appropriate use of technologies such as AR and VR can be employed to develop distinctive cultural tourism experiences. These interactive formats transform abstract cultural concepts into tangible experiences, thereby enhancing visitors' willingness to engage actively. It is worth noting that managers should focus on the process of meaning-making to facilitate the transformation of interactions into attachment. For instance, they can promote visitors' understanding of architecture and culture by offering diverse cultural interpretations and activities, and convey messages through historical memories and local cultural narratives (Cai W. et al., 2025), thereby enabling visitors to engage in deeper cognitive activities.

Secondly, the positive identity climate among residents is crucial for visitors to understand the value of historic buildings and strengthen their emotional connection to the place. Managers can help visitors transform their experiential awareness into attachment by enhancing residents' sense of identity. When residents hold a positive view of their identity, their sense of pride motivates them to participate actively in local preservation and promotion activities. Therefore, managers should actively encourage residents to participate in local revitalisation and preservation efforts. For instance, introducing resident-led tours and storytelling sessions, community cultural festivals, and collaborative activities between residents and visitors can enable tourists to perceive residents' sense of pride through authentic interactions. Providing more opportunities for residents to share their knowledge helps visitors better understand the area's history, culture, and traditional practices (Chan et al., 2024). By sharing residents' stories about the building's history, beliefs, rituals, and events, we construct a shared cultural narrative that communicates the structure's value and significance to the outside world. This helps visitors understand the importance of architectural preservation, fostering a sense of attachment that enhances their intention to engage in destination-supportive behavior.

Furthermore, research findings indicate that the stronger the place attachment tourists develop toward heritage sites, the more likely they are to exhibit supportive behavior intentions toward the destination. If managers integrate heritage tourism with community interests—reinvesting a portion of tourism revenues back into the community to enhance quality of life and cultural preservation (Brooks et al., 2023)—and strengthen community engagement and empowerment through shared responsibility and benefit mechanisms (Li and Hunter, 2015), they can boost resident participation and cultural transmission. This, in turn, fosters more profound visitor attachment to the locality. At the same time, during the process of participation and transmission, visitors' positive feedback also reinforces residents' sense of pride and belonging, fostering a positive and sustainable identity climate. This indicates that if heritage site management adopts enhancing visitors' place attachment as a tourism development strategy, creating a virtuous cycle between visitors and residents through management practices can contribute to the long-term sustainability of the destination's tourism industry.

Finally, based on the findings of this study and the unique characteristics of residential buildings such as the Official-style Mansion, managers should pay attention to shared tourism spaces used by both residents and visitors. Previous research has found that within private residences, visitors are more likely to interact with residents and gain a deeper understanding of local culture (Nguyen et al., 2025). Therefore, as a unique tourism resource, residential areas can be considered as objects of joint participation by managers and residents. Integrating tourism demands with local culture may involve providing more meaningful and in-depth interaction opportunities to cultivate visitors' place attachment, while simultaneously avoiding the limitations of a singular commercial tourism model. Consequently, managers should foster this locally distinctive tourism model through cross-departmental collaboration and joint promotion with residents. This approach may serve as an effective strategy for the sustainable development of heritage tourism.

### 6.2 Theoretical perspective

This study adopts an interdisciplinary approach to integrate embodied cognition theory, identity theory, and the Cognitive-Emotional-Behavioral (CEB) theory, constructing the ECEB-M model to expand and refine the application of the CEB framework within heritage site contexts. The study emphasizes the importance of cognition for place attachment and supportive behavior intention within the context of tourists' embodied experiences. It incorporates residents' identity climate as a social moderator variable in the cognitive-affective stage, examining its influence on the mediating effect strength of embodied cognition-place attachment. This expands the applicability of these theories within the field of heritage tourism and further underscores the pivotal role of social factors in fostering place attachment.

This study expands upon existing research through a review of prior academic literature. First, previous place attachment studies have predominantly focused on robust links between place attachment and behavior, with relatively limited exploration of its antecedent mechanisms. This research adopts an embodied visitor perspective to measure cognitive antecedents associated with historical buildings at heritage sites. Second, previous literature on embodied cognition has primarily focused on specific aspects such as sensory perception, memory, engagement, and meaning, resulting in fragmented research that lacks systematic exploration. Moreover, the system's exploration of this concept has primarily been interpretive and descriptive in nature. Research on the application of embodied cognition systems in heritage tourism, particularly quantitative studies, remains scarce. This study systematically identifies the distinct dimensions of embodied cognition, refines them using the “Cognitive-Emotional-Behavioral” framework, and employs structural equation modeling for quantitative analysis to test the ECEB-M model. This addresses the gap in quantitative research on embodied cognition within heritage tourism while establishing a conceptual model for further evaluation. Third, existing research indicates that psychological connections between residents and visitors significantly influence emotional and behavioral responses, yet their potential as moderating variables within place attachment mechanisms remains under-explored. Therefore, this study highlights the moderating effect of resident identity climate on place attachment mechanisms during visitor cognition, addressing a gap in the literature and further expanding the application boundaries of place attachment and embodied cognition within heritage studies.

## 7 Limitations and future research

This study's findings remain subject to certain limitations. First, the study of urban architecture is limited to Official-style Mansions within Quanzhou's historic district. While these structures are highly representative, the analysis lacks consideration of other heritage sites and building types. Furthermore, heritage sites in different regions and cultural contexts may exhibit variations in management models and regulatory frameworks, which could potentially limit the external validity of the conclusions. Therefore, future research could be extended to different types of heritage sites to enhance the external validity of the findings. Secondly, the sample scope was limited to visitors in the historic district of Quanzhou, primarily comprising local and domestic tourists; results for overseas visitors may differ. Future research could differentiate visitor types and, by incorporating objective indicators, further explore emotional and behavioral differences among distinct groups. Finally, this study only examined resident identity climate as a social moderator. Future research may broaden its scope to include additional potential social moderators, such as community cohesion and trust, to gain a more comprehensive understanding of the mechanisms underlying place attachment formation.

## Data Availability

The original contributions presented in the study are included in the article/supplementary material, further inquiries can be directed to the corresponding author.

## References

[B1] Agapito D. Mendes J. Valle P. (2013b). Exploring the conceptualization of the sensory dimension of tourist experiences. J. Destination Mark. Manage. 2, 62–73. doi: 10.1016/j.jdmm.2013.03.001

[B2] Agapito D. Oom do Valle P. da Costa Mendes J. (2013a). The cognitive-affective-conative model of destination image: a confirmatory analysis. J. Travel Tourism Mark. 30, 471–481. doi: 10.1080/10548408.2013.803393

[B3] Aleshinloye K. D. Woosnam K. M. Joo D. (2024). The influence of place attachment and emotional solidarity on residents' involvement in tourism: perspectives from Orlando, Florida. J. Hospitality Tour. Insights 7, 914–931. doi: 10.1108/JHTI-09-2023-0664

[B4] Anton C. E. Lawrence C. (2014). Home is where the heart is: the effect of place of residence on place attachment and community participation. J. Environ. Psychol. 40, 451–461. doi: 10.1016/j.jenvp.2014.10.007

[B5] Ashforth B. (2000). Role Transitions in Organizational Life: An Identity-Based Perspective. New York, NY: Routledge. doi: 10.4324/9781410600035

[B6] Barclay D. Higgins C. Thompson R. (1995). The Partial Least Squares (PLS) approach to causal modeling: personal computer adoption and use as an illustration. Technol. Stud. 2, 285–309.

[B7] Borghi A. M. Cimatti F. (2010). Embodied cognition and beyond: acting and sensing the body. Neuropsychologia 48, 763–773. doi: 10.1016/j.neuropsychologia.2009.10.029 19913041

[B8] Breakwell G. M. Fife-Schaw C. Lee T. Spencer J. (1986). Attitudes to new technology in relation to social beliefs and group memberships: a preliminary investigation. Curr. Psychol. Res. Rev. 5, 34–47. doi: 10.1007/BF02686595

[B9] Bricker K. S. Kerstetter D. L. (2000). Level of specialization and place attachment: an exploratory study of whitewater recreationists. Leisure Sci. 22, 233–257. doi: 10.1080/01490409950202285

[B10] Brochado A. Stoleriu O. Lupu C. (2021). Wine tourism: a multisensory experience. Curr. Issues Tourism 24, 597–615. doi: 10.1080/13683500.2019.1649373

[B11] Brooks C. Waterton E. Saul H. Renzaho A. (2023). Exploring the relationships between heritage tourism, sustainable community development and host communities' health and wellbeing: a systematic review. PLoS ONE 18:e0282319. doi: 10.1371/journal.pone.0282319 36989275 PMC10058118

[B12] Buonincontri P. Marasco A. Ramkissoon H. (2017). Visitors' experience, place attachment and sustainable behaviour at cultural heritage sites: a conceptual framework. Sustainability 9:1112. doi: 10.3390/su9071112

[B13] Cai W. Shu Z. Liu Y. (2025). Exploring the role of place attachment in shaping sustainable behaviors towards marine cultural heritage: a case study of dongmen village in Fujian Province, China. Front. Psychol. 16:1476308. doi: 10.3389/fpsyg.2025.1476308 39950078 PMC11824459

[B14] Cai X. Hu Z. He J. Zou X. Morrison A. M. (2025). A meta-analysis of the antecedents and outcomes of tourist place attachment. J. Hospitality Tourism Manage. 64:101256. doi: 10.1016/j.jhtm.2025.02.006

[B15] Canepa E. (2022). Architecture is Atmosphere: Notes on Empathy, Emotions, Body, Brain, and Space. Milan: Mimesis international.

[B16] Cao Y. Qin X. Li J. Long Q. Hu B. (2022). Exploring seniors' continuance intention to use mobile social network sites in China: a cognitive-affective-conative model. Univ. Access Inf. Soc. 21, 71–92. doi: 10.1007/s10209-020-00762-3

[B17] Chan S. H. G. Lee W. H. H. Tang B. M. Chen Z. (2024). Legacy of culture heritage building revitalization: place attachment and culture identity. Front. Psychol. 14:1314223. doi: 10.3389/fpsyg.2023.1314223 38333428 PMC10851752

[B18] Chen H. Rahman I. (2018). Cultural tourism: an analysis of engagement, cultural contact, memorable tourism experience and destination loyalty. Tourism Manage. Perspect. 26, 153–163. doi: 10.1016/j.tmp.2017.10.006

[B19] Chen W. Li T. Zhang Y. (2025). Embodied cognition model for museum gamification cultural heritage communication a grounded theory study. Npj Heritage Sci. 13:239. doi: 10.1038/s40494-025-01821-9

[B20] Cheng T. M. Wu H. C. (2015). How do environmental knowledge, environmental sensitivity, and place attachment affect environmentally responsible behavior? An integrated approach for sustainable island tourism. J. Sustain. Tourism 23, 557–576. doi: 10.1080/09669582.2014.965177

[B21] Cheng W. Wang Z. Chen Q. (2021). Tourism environment fit, place attachment, and pro-environmental behavior of tourists: a case study of Wulingyuan World Heritage Site. Resour. Environ. Yangtze Basin 30, 1879–1889.

[B22] Cittati V. M. Balest J. Exner D. (2022). What is the relationship between collective memory and the commoning process in historical building renovation projects? the case of the mas di sabe, Northern Italy. Sustainability 14:11870. doi: 10.3390/su141911870

[B23] Clark A. (1999). An embodied cognitive science? Trends Cognit. Sci. 3, 345–351. doi: 10.1016/S1364-6613(99)01361-3 10461197

[B24] Cohen J. (1988). Statistical Power Analysis for the Behavioral Sciences (2nd edn.). Hillsdale, NJ: Lawrence Erlbaum Associates.

[B25] Corstorphine E. (2006). Cognitive-emotional-behavioural therapy for the eating disorders: working with beliefs about emotions. Eur. Eating Disord. Rev. Prof. J. Eating Disord. Assoc. 14, 448–461. doi: 10.1002/erv.747

[B26] Daryanto A. Song Z. (2021). A meta-analysis of the relationship between place attachment and pro-environmental behaviour. J. Bus. Res. 123, 208–219. doi: 10.1016/j.jbusres.2020.09.045

[B27] Dash G. Paul J. (2021). CB-SEM vs PLS-SEM methods for research in social sciences and technology forecasting. Technol. Forecast. Soc. Change 173:121092. doi: 10.1016/j.techfore.2021.121092

[B28] Davis P. Huang H. Y. Liu W. C. (2010). Heritage, local communities and the safeguarding of ‘Spirit of Place' in Taiwan. Museum Soc. 8, 80–89. doi: 10.29311/mas.v8i2.155

[B29] Deng Y. Zhang X. Zhang B. Zhang B. Qin J. (2023). From digital museuming to on-site visiting: the mediation of cultural identity and perceived value. Front. Psychol. 14:1111917. doi: 10.3389/fpsyg.2023.1111917 37034942 PMC10074853

[B30] Dlamini S. Tesfamichael S. G. Breetzke G. D. Mokhele T. (2021). Spatio-temporal patterns and changes in environmental attitudes and place attachment in Gauteng, South Africa. Geo-spatial Inf. Sci. 24, 666–677. doi: 10.1080/10095020.2021.1976599

[B31] Dongnan News. (2024). Quanzhou 2024nian Jiedai Youkeliang Tupo 1 Yi Renci, Wu Xinchun Wenlv Xiaofei Ji Huodong Tuigao Jieri Redu [泉州2024年接待游客量突破1亿人次,无新春文旅消费季活动推高节日热度]. Dongnan News. Available online at: https://qz.fjsen.com/2025-01/14/content_31822409.htm (Accessed July 31, 2025).

[B32] Eusébio C. Vieira A. L. Lima S. (2018). Place attachment, host-tourist interactions, and residents' attitudes towards tourism development: the case of Boa Vista Island in C ape Verde. J. Sustain. Tourism 26, 890–909. doi: 10.1080/09669582.2018.1425695

[B33] Fan D. X. Zhang H. Q. Jenkins C. L. Lin P. M. (2017). Does tourist-host social contact reduce perceived cultural distance? J. Travel Res. 56, 998–1010. doi: 10.1177/0047287517696979

[B34] Fei H. T. U. Chang C. I. Ward R. F. (1946). Earthbound China: A Study of Rural Economy in Yunnan. London: Routledge.

[B35] Foglia L. Wilson R. A. (2013). Embodied cognition. Wiley Interdiscip Rev Cogn Sci. 4, 319–325. doi: 10.1002/wcs.1226 26304209

[B36] Fornell C. Larcker D. F. (1981). Evaluating structural equation models with unobservable variables and measurement error. J. Mark. Res. 18, 39–50. doi: 10.1177/002224378101800104

[B37] Gagné R. M. (1985). The Conditions of Learning and Theory of Instruction. New York, NY: Holt, Rinehart and Winston.

[B38] Gefen D. Straub D. Boudreau M. C. (2000). Structural equation modeling and regression: guidelines for research practice. Commun. Assoc. Inf. Syst. 4:7. doi: 10.17705/1CAIS.00407

[B39] Gieryn T. F. (2000). A space for place in sociology. Ann. Rev. Sociol. 26, 463–496. doi: 10.1146/annurev.soc.26.1.463

[B40] Goulding C. Domic D. (2009). Heritage, identity and ideological manipulation: the case of Croatia. Ann. Tourism Res. 36, 85–102. doi: 10.1016/j.annals.2008.10.004

[B41] Gu H. Ryan C. (2008). Place attachment, identity and community impacts of tourism-the case of a Beijing hutong. Tourism Manage. 29, 637–647. doi: 10.1016/j.tourman.2007.06.006

[B42] Gursoy D. Zhang C. Chi O. H. (2019). Determinants of locals' heritage resource protection and conservation responsibility behaviors. Int. J. Contemp. Hospitality Manage. 31, 2339–2357. doi: 10.1108/IJCHM-05-2018-0344

[B43] Hair J. F. Hult G. T. M. Ringle C. M. Sarstedt M. Thiele K. O. (2017). Mirror, mirror on the wall: a comparative evaluation of composite-based structural equation modeling methods. J. Acad. Mark. Sci. 45, 616–632. doi: 10.1007/s11747-017-0517-x

[B44] Hair J. F. Risher J. J. Sarstedt M. Ringle C. M. (2019). When to use and how to report the results of PLS-SEM. Eur. Bus. Rev. 31, 2–24.

[B45] Hair J. F. Sarstedt M. Ringle C. M. Mena J. A. (2012). An assessment of the use of partial least squares structural equation modeling in marketing research. J. Acad. Mark. Sci. 40, 414–433. doi: 10.1007/s11747-011-0261-6

[B46] Halpenny E. A. (2010). Pro-environmental behaviours and park visitors: the effect of place attachment. J. Environ. Psychol. 30, 409–421. doi: 10.1016/j.jenvp.2010.04.006

[B47] Hawke S. K. (2010). Belonging: the contribution of heritage to sense of place. Heritage 1, 1331–1339.

[B48] Hayes M. (2020). The coloniality of UNESCO's heritage urban landscapes: heritage process and transnational gentrification in Cuenca, Ecuador. Urban Stud. 57, 3060–3077. doi: 10.1177/0042098019888441

[B49] Heidegger M. (1977). Basic Writings: from Being and Time (1927) to The Task of Thinking (1964). New York, NY: HarperCollins.

[B50] Hilditch D. J. (1995). At the Heart of the World: Merleau-Ponty and the Existential Phenomenology of Embodied and Embedded Intelligence in Everyday Coping. St. Louis, MO: Washington University in St. Louis.

[B51] Hirschman E. C. Holbrook M. B. (1986). “Expanding the ontology and methodology of research on the consumption experience,” in Perspectives on Methodology in Consumer Research (New York, NY: Springer New York), 213–251. doi: 10.1007/978-1-4613-8609-4_7

[B52] Holland O. (2004). “The future of embodied artificial intelligence: machine consciousness?,” in Embodied Artificial Intelligence: International Seminar, Dagstuhl Castle, Germany, July 7-11, 2003. Revised Papers (Berlin, Heidelberg: Springer Berlin Heidelberg), 37–53. doi: 10.1007/978-3-540-27833-7_3

[B53] Horlacher P. (2024). “Tourists go home: stakeholder attitudes in the face of overtourism the case of Dubrovnik, Croatia,” in ISCONTOUR 2024 Tourism Research Perspectives: Proceedings of the International Student Conference in Tourism Research (Norderstedt: BoD-Books on Demand), 276–287.

[B54] Hosany S. Gilbert D. (2010). Measuring tourists' emotional experiences toward hedonic holiday destinations. J. Travel Res. 49, 513–526.

[B55] Hu H. Li Q. Cao X. (2025). Meth research on design paradigm of cultural heritage based on embodied cognition. Int. J. Hum. Comp. Interact. 41, 1860–1871. doi: 10.1080/10447318.2022.2087268

[B56] Huang T. Zhang Y. (2024). 'Roots' tourists' personal heritage experience: an extended cognitive-affective-conative model. J. Hospitality Tourism Manage. 61, 212–223. doi: 10.1016/j.jhtm.2024.10.009

[B57] Huang X. Yang W. (2014). Structural analysis of place attachment in tourist destinations based on confirmatory factor analysis: a case study of Baiyun Mountain in Guangzhou. Hum. Geogr. 29, 144–149.

[B58] Hunter W. C. (2011). Rukai indigenous tourism: representations, cultural identity and Q method. Tourism Manage. 32, 335–348. doi: 10.1016/j.tourman.2010.03.003

[B59] Jelić A. Staničić A. (2022). Embodiment and meaning-making: interdisciplinary perspectives on heritage architecture. J. Archit. 27, 473–484. doi: 10.1080/13602365.2022.2132769

[B60] Jiaxing L. Yongchao Z. Liu J. Pohsun W. (2025). Unraveling tourist behavioral intentions in historic urban built environment: the mediating role of perceived value via SOR MODEL in Macau's heritage sites. Buildings 15:2316. doi: 10.3390/buildings15132316

[B61] Kastenholz E. Carneiro M. J. Eusébio C. Figueiredo E. (2013). Host-guest relationships in rural tourism: evidence from two Portuguese villages. Anatolia 24, 367–380. doi: 10.1080/13032917.2013.769016

[B62] Kim J. H. Ritchie J. B. McCormick B. (2012). Development of a scale to measure memorable tourism experiences. J. Travel Res. 51, 12–25. doi: 10.1177/0047287510385467

[B63] Kim K. Wang Y. Shi J. Guo W. Zhou Z. Liu Z. . (2023). Structural relationship between ecotourism motivation, satisfaction, place attachment, and environmentally responsible behavior intention in nature-based camping. Sustainability 15:8668. doi: 10.3390/su15118668

[B64] Kim M. Lee G. (2022). The effect of servicescape on place attachment and experience evaluation: the importance of exoticism and authenticity in an ethnic restaurant. Int. J. Contemp. Hospitality Manage. 34, 2664–2683. doi: 10.1108/IJCHM-07-2021-0929

[B65] Kim Y. H. Kim D. J. Wachter K. (2013). A study of mobile user engagement (MoEN): engagement motivations, perceived value, satisfaction, and continued engagement intention. Decis. Supp. Syst. 56, 361–370. doi: 10.1016/j.dss.2013.07.002

[B66] Kock F. Ringberg T. (2019). Embodied cognition effects on tourist behavior. Ann. Tourism Res. 78:1. doi: 10.1016/j.annals.2019.05.002

[B67] Kyle G. Graefe A. Manning R. (2005). Testing the dimensionality of place attachment in recreational settings. Environ. Behav. 37, 153–177. doi: 10.1177/0013916504269654

[B68] Lee T. H. (2011). How recreation involvement, place attachment and conservation commitment affect environmentally responsible behavior. J. Sustainable Tourism 19, 895–915. doi: 10.1080/09669582.2011.570345

[B69] Leitan N. Chaffey L. (2014). Embodied cognition and its applications: a brief review. Sensoria J. Mind Brain Cult. 10, 3–10. doi: 10.7790/sa.v10i1.384

[B70] Levent N. Pascual-Leone A. (2014). The Multisensory Museum: Cross-Disciplinary Perspectives on Touch, Sound, Smell, Memory, and Space. Lanham, MD: Rowman & Littlefield. doi: 10.5040/9798881816100

[B71] Lewicka M. (2011). Place attachment: how far have we come in the last 40 years? J. Environ. Psychol. 31, 207–230. doi: 10.1016/j.jenvp.2010.10.001

[B72] Li X. Ma Z. Wang S. (2025). Understanding users' recommendation intention of online museums: a perspective of the cognition-emotion-behavior theory and the expectation confirmation model. npj Heritage Sci. 13:99. doi: 10.1038/s40494-025-01578-1

[B73] Li Y. Hunter C. (2015). Community involvement for sustainable heritage tourism: a conceptual model. J. Cult. Heritage Manage. Sustainable Dev. 5, 248–262. doi: 10.1108/JCHMSD-08-2014-0027

[B74] Lin L. Huang Z. Othman B. Luo Y. (2020). Let's make it better: an updated model interpreting international student satisfaction in China based on PLS-SEM approach. PLoS ONE 15:e0233546. doi: 10.1371/journal.pone.0242583 32628675 PMC7337283

[B75] Liu Y. Minamikawa K. (2024). From sensory experience to revisit intentions: an embodied cognition perspective on replica tourism. Sustainability 16:8030. doi: 10.3390/su16188030

[B76] Malliet S. (2017). Interactive digital narrative: history, theory and practice. Communications: the European Journal of Communication Research/Deutsche Gesellschaft für Kommunikationsforschung [Köln]. 42, 104–106.

[B77] Martínez Yáñez C. Maclaren F. Smith-Christensen C. Gowen M. Donovan J. Kelly I. . (2022). “Reinforcing cultural heritage protection and community resilience through responsible and sustainable tourism management,” in Adopted by the ICOMOS Annual General Assembly (Bangkok, Thailand) in November 2022 = *ICOMOS International Charter for Cultural Heritage Tourism (2022): styrkelse af kulturarvsbeskyttelse og samfundsresiliens gennem ansvarlig og bæredygtig turismeforvaltning Vedtaget af ICOMOS Årlige Generalforsamling* (Bangkok).

[B78] McIlvenny P. Broth M. Haddington P. (2009). Communicating place, space and mobi lity. J. Pragmatics 41, 1879–1886. doi: 10.1016/j.pragma.2008.09.014

[B79] McIntosh A. J. (1999). Into the tourist's mind: understanding the value of the heritage experience. J. Travel Tourism Mark. 8, 41–64. doi: 10.1300/J073v08n01_03

[B80] Merleau-Ponty M. Landes D. Carman T. Lefort C. (2013). Phenomenology of Perception. London: Routledge. doi: 10.4324/9780203720714

[B81] Mikulić J. Ryan C. (2018). Reflective versus formative confusion in SEM based tourism research: a critical comment. Tourism Manage. 68, 465–469. doi: 10.1016/j.tourman.2018.05.002

[B82] Mistry J. Berardi A. Tschirhart C. Bignante E. Haynes L. Benjamin R. . (2015). Indigenous identity and environmental governance in Guyana, South America. Cult. Geographies 22, 689–712. doi: 10.1177/1474474014560998

[B83] Monterrubio J. C. Mendoza-Ontiveros M. M. (2014). Tourism and the demonstration effect: empirical evidence. Tourism Manage. Stud. 10, 97–103.

[B84] Naseri S. A. (2020). Effect of destination image on revisit intention and environmentally responsible behavior through tourist satisfaction in Famagusta city of north Cyprus (master's thesis). Eastern Mediterranean University (EMU)-Dogu Akdeniz Üniversitesi (DAÜ)), Famagusta, Cyprus.

[B85] Nguyen K. T. Murphy L. Chen T. (2025). The influence of host-tourist interaction on visitor perception of long-term ethnic tourism outcomes: considering a complexity of physical settings. J. Hospitality Tourism Manage. 62, 304–320. doi: 10.1016/j.jhtm.2025.02.004

[B86] Nguyen K. T. Murphy L. Chen T. Pearce P. L. (2024). Let's listen: the voices of ethnic villagers in identifying host-tourist interaction issues in the Central Highlands, Vietnam. J. Heritage Tourism 19, 263–286. doi: 10.1080/1743873X.2023.2259512

[B87] Niedenthal P. M. (2007). Embodying emotion. Science 316, 1002–1005. doi: 10.1126/science.1136930 17510358

[B88] Niedenthal P. M. Brauer M. Halberstadt J. B. Innes-Ker Å. H. (2001). When did her smile drop? Facial mimicry and the influences of emotional state on the detection of change in emotional expression. Cognit. Emotion 15, 853–864. doi: 10.1080/02699930143000194

[B89] Nunkoo R. Gursoy D. (2012). Residents' support for tourism: an identity perspective. Ann. Tourism Res. 39, 243–268. doi: 10.1016/j.annals.2011.05.006

[B90] Omo-Obas P. Anning-Dorson T. (2023). Cognitive-affective-motivation factors influencing international visitors' destination satisfaction and loyalty. J. Hospitality Tourism Insights 6, 2222–2240. doi: 10.1108/JHTI-05-2022-0178

[B91] Oorgaz-Agüera F. Puig-Cabrera M. Moral-Cuadra S. Domínguez-Valerio C. M. (2025). Authenticity of architecture, place attachment, identity and support for sustainable tourism in world heritage cities. Tourism Hospitality Manage. 31, 81–92. doi: 10.20867/thm.31.1.6

[B92] O'regan J. K. Noë A. (2001). A sensorimotor account of vision and visual consciousness. Behav. Brain Sci. 24, 939–973. doi: 10.1017/S0140525X01000115 12239892

[B93] Ozolins U. Hale S. Cheng X. Hyatt A. Schofield P. (2020). Translation and back-translation methodology in health research-a critique. Expert Review Pharmacoecon. Outcomes Res. 20, 69–77. doi: 10.1080/14737167.2020.1734453 32089017

[B94] Palmer C. (1999). Tourism and the symbols of identity. Tourism Manage. 20, 313–321. doi: 10.1016/S0261-5177(98)00120-4

[B95] Pandža Bajs I. (2015). Tourist perceived value, relationship to satisfaction, and behavioral intentions: the example of the Croatian tourist destination Dubrovnik. J. Travel Res. 54, 122–134. doi: 10.1177/0047287513513158

[B96] Peng J. Yang X. Fu S. Huan T. C. T. (2023). Exploring the influence of tourists' happiness on revisit intention in the context of traditional Chinese medicine cultural tourism. Tourism Manage. 94:104647. doi: 10.1016/j.tourman.2022.104647

[B97] Peng X. Liu M. Hu Q. He X. (2023). A multiscale perspective on place attachment and pro-environmental behavior in hotel spaces. J. Hospitality Tourism Manage. 55, 435–447. doi: 10.1016/j.jhtm.2023.05.013

[B98] People's Daily. (2024). Zanhua You Gucuo, Pincha Shang Nanyin: Fujian Quanzhou Tuidong Wenlv Ronghe [簪花游古厝 品茶赏南音:福建泉州推动文旅融合]. People's Daily. Available online at: https://www.quanzhou.gov.cn/zfb/xxgk/zfxxgkzl/qzdt/qzyw/202404/t20240415_3026962.htm (Accessed July 31, 2025).

[B99] Perneger T. V. Courvoisier D. S. Hudelson P. M. Gayet-Ageron A. (2015). Sample size for pre-tests of questionnaires. Qual. Life Res. 24, 147–151. doi: 10.1007/s11136-014-0752-2 25008261

[B100] Pfeifer R. Bongard J. (2006). How the Body Shapes the Way We Think: A New View of Intelligence. Cambridge, MA: MIT Press. doi: 10.7551/mitpress/3585.001.0001

[B101] Proshansky H. M. (1978). The city and self-identity. Environ. Behav. 10, 147–169. doi: 10.1177/0013916578102002

[B102] Qiu N. Wu J. Li H. Pan C. Guo J. (2025). Relationship between perceived authenticity, place attachment, and tourists' environmental behavior in industrial heritage. Sustainability 17:5152. doi: 10.3390/su17115152

[B103] Qu C. Zhang C. Shen S. Olsen D. H. (2023). Heritage conservation and communities' sense of deprivation in tourism: the case of the Hani community in Yunnan, China. Tourism Geographies 25, 881–898. doi: 10.1080/14616688.2021.2016936

[B104] Quynh N. Hoai N. T. Loi N. V. (2021). The role of emotional experience and destination image on ecotourism satisfaction. Spanish J. Mark. ESIC 25, 312–332. doi: 10.1108/SJME-04-2020-0055

[B105] Ramkissoon H. Smith L. D. G. Weiler B. (2013). Relationships between place attachment, place satisfaction and pro-environmental behaviour in an Australian national park. J. Sustain. Tourism 21, 434–457. doi: 10.1080/09669582.2012.708042

[B106] Ramkissoon H. Weiler B. Smith L. D. G. (2012). Place attachment and pro-environmental behaviour in national parks: the development of a conceptual framework. J. Sustain. Tourism 20, 257–276. doi: 10.1080/09669582.2011.602194

[B107] Rehman Khan S. A. Yu Z. (2021). Assessing the eco-environmental performance: an PLS-SEM approach with practice-based view. Int. J. Logist. Res. Appl. 24, 303–321. doi: 10.1080/13675567.2020.1754773

[B108] Ren Q. He B. Chen X. Han J. Han F. (2021). The mechanism and mediating effect of the “perception-emotion-behaviour” chain of tourists at world natural heritage sites-a case study from Bayanbulak, China. Int. J. Environ. Res. Public Health 18:12531. doi: 10.3390/ijerph182312531 34886256 PMC8656651

[B109] Ringle C. M. Sarstedt M. Sinkovics N. Sinkovics R. R. (2023). A perspective on using partial least squares structural equation modelling in data articles. Data Brief 48:109074. doi: 10.1016/j.dib.2023.109074 37066088 PMC10090253

[B110] Roemer E. Schuberth F. Henseler J. (2021). HTMT2-an improved criterion for assessing discriminant validity in structural equation modeling. Ind. Manage. Data Syst. 121, 2637–2650. doi: 10.1108/IMDS-02-2021-0082

[B111] Ross L. Lepper M. Ward A. (2010). “History of social psychology: insights, challenges, and contributions to theory and application,” *Handbook of Social Psychology*, eds S. T. Fiske, D. T. Gilbert and G. Lindzey (Hoboken, NJ: Wiley). doi: 10.1002/9780470561119.socpsy001001

[B112] Rugwiji T. T. (2019). Identity reconstruction of the Great Zimbabwe National Monument: an indigenous knowledge systems perspective. Stud. Historiae Ecclesiasticae 45, 1–18. doi: 10.25159/2412-4265/4145

[B113] Sahabuddin M. Tan Q. Hossain I. Alam M. S. Nekmahmud M. (2021). Tourist environmentally responsible behavior and satisfaction; Study on the world's longest natural sea beach, Cox's Bazar, Bangladesh. Sustainability 13:9383. doi: 10.3390/su13169383

[B114] Scannell L. Gifford R. (2010). Defining place attachment: a tripartite organizing framework. J. Environ. Psychol. 30, 1–10. doi: 10.1016/j.jenvp.2009.09.006

[B115] Shin H. Sharma A. Nicolau J. L. Lee J. (2025). Transformative outcomes of workcation: satisfaction, place attachment, and behavioral intentions. J. Travel Res. doi: 10.1177/00472875251318317

[B116] Sinclair-Maragh G. Gursoy D. (2017). Residents' identity and tourism development: the Jamaican perspective. Int. J. Tourism Sci. 17, 107–125. doi: 10.1080/15980634.2017.1313472

[B117] Sonuç N. (2023). “Culture, tourism, and sustainability (cultural heritage and sustainable tourism, social sustainability of tourism, socio-cultural sustainability of tourism),” in Encyclopedia of Sustainable Management (Cham: Springer International Publishing), 1083–1089. doi: 10.1007/978-3-031-25984-5_457

[B118] Stylidis D. (2022). Exploring resident-tourist interaction and its impact on tourists' destination image. J. Travel Res. 61, 186–201. doi: 10.1177/0047287520969861

[B119] Su X. Li X. Wu Y. Yao L. (2020). How is intangible cultural heritage valued in the eyes of inheritors? Scale development and validation. J. Hospitality Tourism Res. 44, 806–834. doi: 10.1177/1096348020914691

[B120] Sumartojo S. (2022). Approaching heritage sites atmospherically. J. Archit. 27, 558–572. doi: 10.1080/13602365.2022.2122069

[B121] Tan S. K. Tan S. H. (2020). Clan/geographical association heritage as a place-based approach for nurturing the sense of place for locals at a world heritage site. J. Hospitality Tourism Manage. 45, 592–603. doi: 10.1016/j.jhtm.2020.10.017

[B122] Tuan Y. F. (1977). Space and Place: The Perspective of Experience. Minneapolis, MN: University of Minnesota Press.

[B123] Tuan Y. F. (1990). Topophilia: A Study of Environmental Perception, Attitudes, and Values. New York, NY: Columbia University Press.

[B124] Ujang N. Zakariya K. (2015). The notion of place, place meaning and identity in urban regeneration. Proc. Soc. Behav. Sci. 170, 709–717. doi: 10.1016/j.sbspro.2015.01.073

[B125] Vacharkulksemsuk T. Fredrickson B. L. (2012). Strangers in sync: achieving embodied rapport through shared movements. J. Exp. Soc. Psychol. 48, 399–402. doi: 10.1016/j.jesp.2011.07.015 22389521 PMC3290409

[B126] Van Dijk L. Rietveld E. (2017). Foregrounding sociomaterial practice in our understanding of affordances: the skilled intentionality framework. Front. Psychol. 7:1969. doi: 10.3389/fpsyg.2016.01969 28119638 PMC5220071

[B127] van Rekom J. Go F. (2006). Being discovered: A blessing to local identities? Ann. Tour. Res. 33, 767–784.

[B128] Vaske J. J. Kobrin K. C. (2001). Place attachment and environmentally responsible behavior. J. Environ. Educ. 32, 16–21. doi: 10.1080/00958960109598658

[B129] Vignoles V. L. Chryssochoou X. Breakwell G. M. (2002). Evaluating models of identity motivation: self-esteem is not the whole story. Self Identity 1, 201–218. doi: 10.1080/152988602760124847

[B130] Voorhees C. M. Brady M. K. Calantone R. Ramirez E. (2016). Discriminant validity testing in marketing: an analysis, causes for concern, and proposed remedies. J. Acad. Mark. Sci. 44, 119–134. doi: 10.1007/s11747-015-0455-4

[B131] Vygotsky L. S. Cole M. (1978). Mind in Society: Development of Higher Psychological Processes. Cambridge, MA: Harvard University Press.

[B132] Wang S. Xu H. (2015). Influence of place-based senses of distinctiveness, continuity, self-esteem and self-efficacy on residents' attitudes toward tourism. Tourism Manage. 47, 241–250. doi: 10.1016/j.tourman.2014.10.007

[B133] Wang X. Qin X. Zhou Y. (2020). A comparative study of relative roles and sequences of cognitive and affective attitudes on tourists' pro-environmental behavioral intention. J. Sustain. Tourism 28, 727–746. doi: 10.1080/09669582.2019.1704297

[B134] Waterton E. (2014). A more-than-representational understanding of heritage? The 'past'and the politics of affect. Geogr. Compass 8, 823–833. doi: 10.1111/gec3.12182

[B135] Williams D. R. Patterson M. E. Roggenbuck J. W. Watson A. E. (1992). Beyond the commodity metaphor: examining emotional and symbolic attachment to place. Leisure Sci. 14, 29–46. doi: 10.1080/01490409209513155

[B136] Williams D. R. Roggenbuck J. W. (1989). “Measuring place attachment: some preliminary results,” in NRPA Symposium on Leisure Research, San Antonio, TX (Vol. 9) (Virginia: Virginia Polytechnic Institute & State University).

[B137] Williams D. R. Vaske J. J. (2003). The measurement of place attachment: validity and generalizability of a psychometric approach. For. Sci. 49, 830–840. doi: 10.1093/forestscience/49.6.830

[B138] Wilson M. (2002). Six views of embodied cognition. Psychonomic Bull. Rev. 9, 625–636. doi: 10.3758/BF03196322 12613670

[B139] Wu H. C. Li T. (2017). A study of experiential quality, perceived value, heritage image, experiential satisfaction, and behavioral intentions for heritage tourists. J. Hospitality Tourism Res. 41, 904–944. doi: 10.1177/1096348014525638

[B140] Xu S. Kim H. J. Liang M. Ryu K. (2018). Interrelationships between tourist involvement, tourist experience, and environmentally responsible behavior: a case study of Nansha Wetland Park, China. J. Travel Tourism Mark. 35, 856–868. doi: 10.1080/10548408.2018.1439429

[B141] Yang W. Chen Q. Huang X. Xie M. Guo Q. (2022). How do aesthetics and tourist involvement influence cultural identity in heritage tourism? The mediating role of mental experience. Front. Psychol. 13:990030. doi: 10.3389/fpsyg.2022.990030 36389488 PMC9650545

[B142] Yang Y. Bao J. (2012). An analysis of the characteristics of place attachment among non-local business operators in a tourism community: a case study of West Street, Yangshuo. Human Geogr. 27, 81–86.

[B143] Yi X. Fu X. Lin B. Sun J. (2024). Authenticity, identity, self-improvement, and responsibility at heritage sites: the local residents' perspective. Tourism Manage. 102:104875. doi: 10.1016/j.tourman.2023.104875

[B144] Yi X. Lin V. S. Jin W. Luo Q. (2017). The authenticity of heritage sites, tourists' quest for existential authenticity, and destination loyalty. J. Travel Res. 56, 1032–1048. doi: 10.1177/0047287516675061

[B145] Yin Z. Huang A. Wang J. (2023). Memorable tourism experiences' formation mechanism in cultural creative tourism: from the perspective of embodied cognition. Sustainability 15:4055. doi: 10.3390/su15054055

[B146] Yuksel A. Yuksel F. Bilim Y. (2010). Destination attachment: effects on customer satisfaction and cognitive, affective and conative loyalty. Tourism Manage. 31, 274–284. doi: 10.1016/j.tourman.2009.03.007

[B147] Zeithaml V. A. (1988). Consumer perceptions of price, quality, and value: a means-end model and synthesis of evidence. J. Mark. 52, 2–22. doi: 10.1177/002224298805200302

[B148] Zhang C. X. Fong L. H. N. Li S. Ly T. P. (2019). National identity and cultural festivals in postcolonial destinations. Tourism Manage. 73, 94–104. doi: 10.1016/j.tourman.2019.01.013

[B149] Zhang J. Jin L. Pan X. Wang Y. (2024a). Pro-environmental behavior of tourists in ecotourism scenic spots: the promoting role of tourist experience quality in place attachment. Sustainability 16:8984. doi: 10.3390/su16208984

[B150] Zhang J. Zou Y. Li Y. Peng C. Jin D. (2024b). Embodied power: how do museum tourists' sensory experiences affect place identity? J. Hospitality Tourism Manage. 60, 334–346. doi: 10.1016/j.jhtm.2024.08.009

[B151] Zhang R. Smith L. (2019). Bonding and dissonance: rethinking the interrelations among stakeholders in heritage tourism. Tourism Manage. 74, 212–223. doi: 10.1016/j.tourman.2019.03.004

[B152] Zhang S. Pan Y. (2023). Mind over matter: examining the role of cognitive dissonance and self-efficacy in discontinuous usage intentions on pan-entertainment mobile live broadcast platforms. Behav. Sci. 13:254. doi: 10.3390/bs13030254 36975279 PMC10045806

